# Light-activated mitochondrial fission through optogenetic control of mitochondria-lysosome contacts

**DOI:** 10.1038/s41467-022-31970-5

**Published:** 2022-07-25

**Authors:** Kangqiang Qiu, Weiwei Zou, Hongbao Fang, Mingang Hao, Kritika Mehta, Zhiqi Tian, Jun-Lin Guan, Kai Zhang, Taosheng Huang, Jiajie Diao

**Affiliations:** 1 Department of Cancer Biology, University of Cincinnati College of Medicine, Cincinnati, OH 45267 USA; 2 Division of Human Genetics, Cincinnati Children’s Hospital Medical Center, Cincinnati, OH 45229 USA; 3 Department of Biochemistry, School of Molecular and Cellular Biology, University of Illinois at Urbana-Champaign, Urbana, IL 61801 USA; 4 Department of Molecular Genetics, Biochemistry, and Microbiology, University of Cincinnati College of Medicine, Cincinnati, OH 45267 USA; 5 Department of Pediatrics, University at Buffalo, 1001 Main Street, Buffalo, NY 14203 USA

**Keywords:** Optogenetics, Mechanisms of disease, Supramolecular assembly

## Abstract

Mitochondria are highly dynamic organelles whose fragmentation by fission is critical to their functional integrity and cellular homeostasis. Here, we develop a method via optogenetic control of mitochondria–lysosome contacts (MLCs) to induce mitochondrial fission with spatiotemporal accuracy. MLCs can be achieved by blue-light-induced association of mitochondria and lysosomes through various photoactivatable dimerizers. Real-time optogenetic induction of mitochondrial fission is tracked in living cells to measure the fission rate. The optogenetic method partially restores the mitochondrial functions of SLC25A46^−/−^ cells, which display defects in mitochondrial fission and hyperfused mitochondria. The optogenetic MLCs system thus provides a platform for studying mitochondrial fission and treating mitochondrial diseases.

## Abstract (web summary)

Existing methods can lack spatiotemporal accuracy to manipulate dynamic mitochondrial behaviour in live cells. Here the authors report an optogenetic method to control mitochondria-lysosome contacts and induce mitochondrial fission; they use photoactivatable dimerizers including CRY2/CIB and SspB/iLID.

## Introduction

As essential organelles for eukaryotic life, mitochondria play important roles in cellular homeostasis and various cellular functions^1,2^. They provide the vast majority of adenosine triphosphate (ATP) via oxidative phosphorylation (OXPHOS), store metabolites (e.g., calcium, iron, lipids, and protons), biosynthesize active compounds (iron-sulfur clusters), and function as gatekeepers for apoptosis and inflammation pathways^3–6^. Mitochondria are highly dynamic and undergo constant fission and fusion, whose balance is critical to the maintenance of a functional mitochondrial network in a cell. An imbalance in mitochondrial dynamics can cause mitochondrial dysfunctions and disorders^7–10^. Restoring normal mitochondrial dynamics can restore mitochondrial functions and treat the associated diseases, and methods to regulate mitochondrial fusion and fission are highly sought-after.

Mitochondrial fission allows mitochondrial deoxyribonucleic acid (DNA) synthesis and mitochondrial biogenesis^11,12^ and facilitates metabolic adaptation by regulating the mitochondrial number, distribution, and quality, through concerted processes with autophagy, apoptosis, and cell division^11,13–16^. Mitochondrial fragmentation arises from activated fission (DRP1 activity and short OPA1 level increase; DRP1, dynamin-related protein 1; OPA1, optic atrophy 1) and/or impaired fusion (mitofusin (MFN) level or activity, and long OPA1 level decrease)^17,18^. Defects in the mitochondrial fission machinery could result in hyperfused networks and cause mitochondrial diseases. For example, mutations in the mitochondrial fission gene OPA1 cause autosomal dominant optic atrophy, while mutations in mitochondrial fusion genes, such as mitofusin-2 (MFN2), cause overlapping neurodegenerative phenotypes, including axonal peripheral neuropathy (Charcot-Marie-Tooth neuropathy type 2, CMT2)^19,20^. Recessively inherited mutations in SLC25A46 (solute carrier family 25 member 46), which may inhibit mitochondrial fission, can cause childhood-onset optic atrophy, axonal neuropathy, and cerebellar neurodegeneration^21,22^.

Genetic and pharmacological approaches have been widely used to fragment mitochondria^23–25^ but often lack the spatiotemporal accuracy to track dynamic mitochondrial behavior in live cells. Chemicals such as carbonyl cyanide m-chlorophenylhydrazone and carbonyl cyanide 4-(trifluoromethoxy)phenylhydrazone (FCCP) could induce mitochondrial fragmentation by abolishing the mitochondrial membrane proton gradient but may cause mitochondrial damage/dysfunction^26,27^. Meanwhile, these chemical methods prohibit fragmented mitochondria from elongating again. Therefore, a method that can manipulate mitochondrial fission while allowing spatiotemporal controllability and reversibility is demanded.

An attractive strategy to modulate mitochondrial fission is through mitochondria-lysosome contacts (MLCs). MLCs are bidirectional crosstalk between mitochondria and lysosomes that regulates organelle network dynamics. Mitochondria regulate lysosomal dynamics by modulating Rab7 GTP hydrolysis (Rab7, Rab7A, member RAS oncogene family; GTP, guanosine triphosphate) at contact sites, while lysosomes regulate mitochondrial dynamics by marking sites of mitochondrial fission. MLCs recruit TBC1D15 and FIS1 proteins (TBC1D15, TBC1 domain family member 15; FIS1, fission, mitochondrial 1) to induce mitochondrial fission via RAB7 GTP hydrolysis^5,8–10,28^. Therefore, the induction of MLCs could potentially be used to accelerate mitochondrial fission. However, a method to directly regulate MLCs in a reversible and controllable manner in living cells is critical^29–32^.

With high spatiotemporal accuracy, opsin-free optogenetics uses light to control protein–protein interactions in live cells. It has been used to control diverse cellular and organismal functions such as neuronal activity, intracellular signaling, gene expression, cell proliferation, differentiation, migration, apoptosis, and organelle transport^33–36^. Here, we develop an optogenetic MLCs system to manipulate mitochondrial fission. By investigating the fragmentation of mitochondria by the optogenetic method, we demonstrate the system’s spatiotemporal controllability and reversibility and, in turn, use the system to partially restore the mitochondrial functions of SLC25A46^−/−^ cells.

## Results

### Optogenetics can be used to induce mitochondria-lysosome contacts

To develop an optogenetic tool to control MLCs, we opted to use the blue-light-sensitive heterodimerizer, cryptochrome from *Arabidopsis thaliana* (CRY2) and the N-terminal cryptochrome interacting basic-helix-loop-helix (CIB), which undergo reversible association and dissociation without the need for exogenous cofactors^35^. We first confirmed their interaction by a membrane recruitment assay, consistent with previous results (Supplementary Fig. 1 and Movie 1). We verified that our illumination condition (1 h of continuous blue light illumination at 300 μW/cm^2^) induced negligible phototoxicity because cell viability was similar under light and dark (Supplementary Fig. 2).

**Fig. 1:**
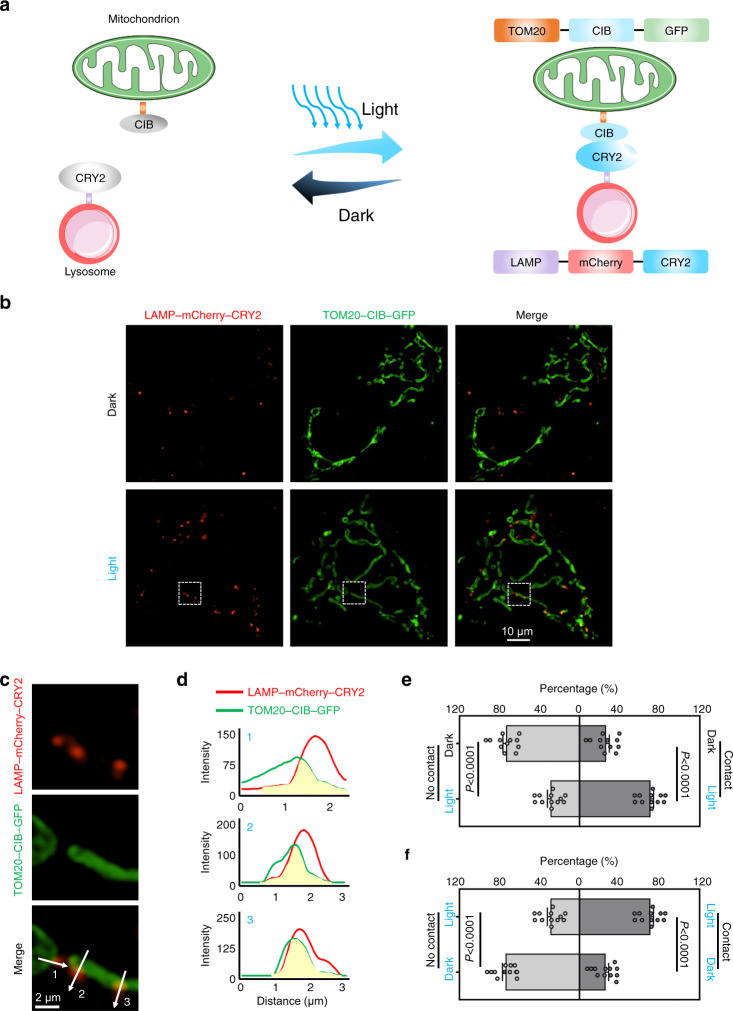
Optogenetics can be used to induce mitochondria-lysosome contacts. **a** Schematic representation of optogenetic induction of MLCs. Light-sensitive proteins CRY2 and CIB are anchored to lysosomes and mitochondria via the specific organelle-targeting transmembrane domains LAMP and TOM20, respectively. Blue light illumination induces CRY2-CIB association and facilitates the formation of MLCs. GFP and mCherry are used as expression markers. **b** Representative structured illumination microscopic images of mitochondria (green) and lysosomes (red) with or without blue light exposure for 20 min at 300 μW/cm^2^. **c** Partially enlarged images of Fig. 1b. **d** Intensities of GFP and mCherry on the white arrows in Fig. 1c. **e** Quantification of percentages of lysosomes contacting and not contacting mitochondria without or with blue light exposure. **f** Quantification of percentages of lysosomes contacting and not contacting mitochondria with blue light exposure or with blue light exposure followed by dark for 24 h. For (**e**) and (**f**), *n* = 12 cells examined over 3 independent experiments. Data are presented as *M* ± *SEM*. The statistical differences between the experimental groups were analyzed by double-tailed Student’s *t* test. When *P* < 0.05, it was considered to have statistical significance. Source data are provided as a Source Data file.

To attach the photoactivatable proteins to the surfaces of mitochondria and lysosomes, we fused the heterodimerizers to fluorescent protein-labeled translocase of outer mitochondrial membrane 20 (TOM20) and lysosomal associated membrane protein 1 (LAMP), generating *LAMP–mCherry–CRY2* and *TOM20–CIB–GFP* (GFP, green fluorescent protein; mCherry, a member of the mFruits family of monomeric red fluorescent proteins; Fig. 1a). We further determined that 1250 ng (for each plasmid, in 240 μL DMEM, 35 mm dish, Supplementary Fig. 3; DMEM, Dulbecco’s modified eagle medium) is the optimal concentration for transfection using structured illumination microscopy (SIM), which overcomes the diffraction limit (<200 nm) of conventional optical microscopes and allows super-resolution imaging at the subcellular level^37^. To investigate MLCs, we exposed the transfected HeLa cells (cervical cancer cells) to blue light (Fig. 1b–d). MLCs visibly increased after blue light illumination, quantified by counting the percentage of effective MLCs sites. The percentage of MLCs increased from 26.46% in the dark to 70.83% under blue light (300 μW/cm^2^, 20 min) (Fig. 1e and Supplementary Fig. 4, 5a). Furthermore, optogenetically induced MLCs were reversible, as the interacting organelles separated in the dark (Fig. 1f and Supplementary Fig. 4, 5b, 6). Together, the results suggest that the optogenetic system reversibly induces MLCs in living cells.

### The optogenetic MLCs system induces reversible mitochondrial fission

**Fig. 2:**
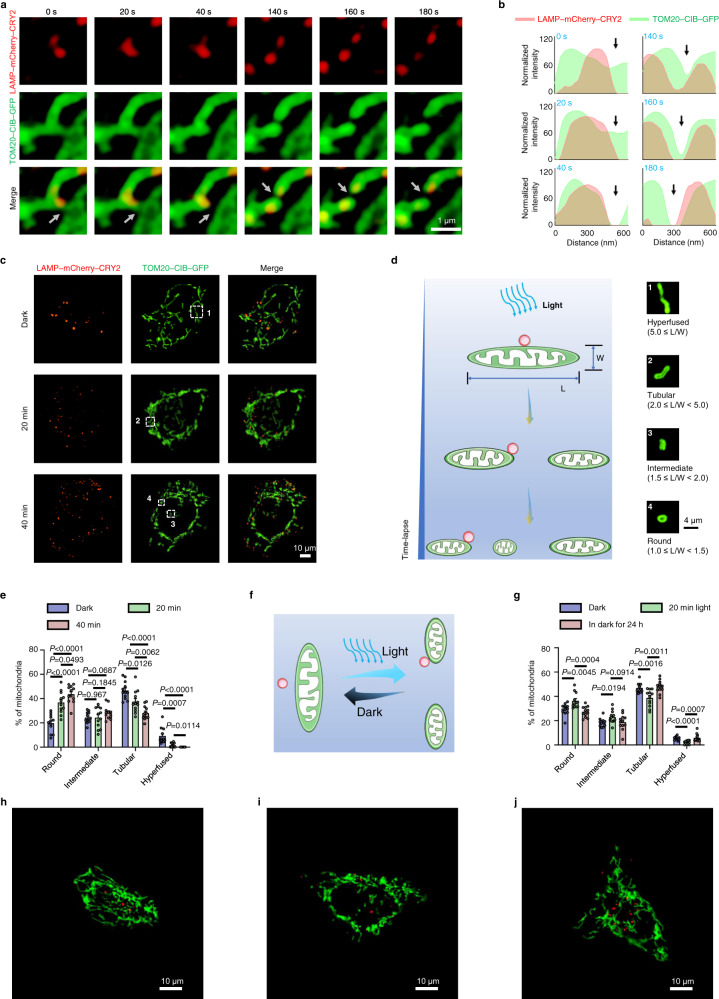
The optogenetic MLCs system induces reversible mitochondrial fission. **a** Representative time-lapse images of lysosomes contacting mitochondria at the site of mitochondrial division (white arrows) during mitochondrial fission in living HeLa cells expressing *LAMP–mCherry–CRY2* (i.e., for lysosomes) and *TOM20–CIB–GFP* (i.e., for mitochondria) under blue light illumination. **b** Line scan corresponding to Fig. 2a showing mitochondrial fission (black arrows) by light-induced MLCs. **c** Representative images of lysosomes and mitochondria under different durations of blue light exposure (0, 20, and 40 min) in living HeLa cells expressing *LAMP–mCherry–CRY2* and *TOM20–CIB–GFP*. **d** Schematic representation of mitochondrial fragmentation induced by MLCs under time-lapse blue light exposure. “L” stands for length; “W” stands for width; “L/W” stands for dividing the length by the width. **e** Quantitative analysis of mitochondrial morphology for HeLa cells expressing *LAMP–mCherry–CRY2* and *TOM20–CIB–GFP* under different durations of blue light exposure. **f** Schematic representation of the reversibility of mitochondrial fission caused by light-induced MLCs. **g** Quantitative analysis of mitochondrial morphology for the reversibility of mitochondrial fission induced by optogenetic MLCs system in living HeLa cells expressing *LAMP–mCherry–CRY2* and *TOM20–CIB–GFP*. Blue: before blue-light exposure; Green: after 20 min blue-light exposure; Pink: after 20 min light illumination then dark for 24 h. **h**–**j** The reversibility of mitochondrial fission induced by optogenetic MLCs system. **h** Before blue-light exposure. **i** After 20 min blue-light expose. **j** After 20 min blue-light exposure then dark for 24 h. For (**e**) and (**g**), *n* = 12 cells examined over 3 independent experiments. Data are presented as *M* ± *SEM*. The statistical differences between the experimental groups were analyzed by double-tailed Student’s *t* test. When *P* < 0.05, it was considered to have statistical significance. Source data are provided as a Source Data file.

MLCs regulate mitochondrial fission, an event that takes only 10 s at 37 °C^28^. However, SIM generally requires more than 10 s to capture an image with a green channel and a red channel. Thus, to track optogenetically-induced mitochondrial fission in real time with SIM, we performed the imaging experiment at room temperature (20 °C) to decelerate cellular activity and set the time interval to 20 s. This way, we successfully captured real-time mitochondrial fission caused by light-induced MLCs (Fig. 2a, Supplementary Movie 2 and 3 and Figs. 7–9). A lysosome moved alongside the fission site during mitochondrial fission until the mitochondrion divided into two sister mitochondria. The overlap of the lysosome and a sister mitochondrion then gradually decreased and finally disappeared (Fig. 2a, b).

We recorded mitochondrial morphology during different intervals of blue light exposure in living HeLa cells. In the control group without light exposure, most mitochondria remained elongated. Under blue light illumination, the proportion of long mitochondria decreased together with an increase of short mitochondria (Fig. 2c, d). To quantify the mitochondrial morphology, we calculated the aspect ratio of each mitochondrion by dividing its length (L) by its width (W) and grouped mitochondria into four categories: round (1.0 ≤ L/W < 1.5), intermediate (1.5 ≤ L/W < 2.0), tubular (2.0 ≤ L/W < 5.0), and hyperfused (5.0 ≤ L/W). Blue light exposure increased the percentage of round mitochondria and decreased the percentages of hyperfused and tubular mitochondria (Fig. 2e, Supplementary Fig. 10 and 11, and Table 1). When cells were kept in the dark for 24 hours after blue light illumination, the fragmented mitochondria became more elongated again (Fig. 2f–j and Supplementary Fig. 12), indicating that the change of mitochondria morphology was reversible following optogenetic treatment. By covering half of a culture dish with tin foil paper under blue light exposure, we demonstrated the ability of spatial control for our optogenetic system (Supplementary Fig. 13). Using a transmission electron microscope, we also confirmed the morphological change of mitochondria upon blue light exposure in transfected HeLa cells (Supplementary Fig. 14).

To ensure that the observed mitochondrial fragmentation resulted from light-mediated MLCs, we performed control experiments without photoactivatable proteins. No noticeable changes in mitochondrial morphology were observed (Supplementary Fig. 15–17), demonstrating that light and photoactivatable proteins were essential factors for increasing mitochondrial fission. In addition, lysosomal targeting is essential for MLCs because no mitochondrial fragmentation was observed in cells co-transfected with *CRY2olig-mCherry* (i.e., no targeting to lysosomes; CRY2olig, CRY2PHR E490G) and *TOM20-CIB-GFP* (Supplementary Fig. 18). Optogenetic induction of MLCs remained effective after switching CRY2 and CIB (i.e., *TOM20-CRY2-GFP* and *LAMP-mCherry-CIB*) (Supplementary Fig. 19 and 20). To further confirm that optogenetic MLCs were not specific to CRY2-CIB interaction, we modified the system to include CRY2 homo-association (*LAMP-mCherry-CRY2* + *TOM20-CRY2-GFP*) (Supplementary Fig. 21), as well as another heterodimerizer SspB-iLID (*TOM20-SspB-GFP* + *LAMP-mCherry-iLID*; SspB, stringent starvation protein B; iLID, improved light inducible dimer). In both cases, blue light enhanced mitochondrial fission similar to that induced by the CRY2/CIB system (Supplementary Fig. 22–23). Besides the HeLa cell line, optogenetic MLCs also enhanced mitochondrial fission in PC12 cells (Supplementary Fig. 24 and Table 2; PC12, rat pheochromocytoma). Therefore, the optogenetic MLCs system efficiently induces mitochondrial fission with reversibility and spatiotemporal controllability.

### The optogenetic MLCs system reduces mitochondrial hyperfusion and restores mitochondrial function in SLC25A46^−/−^ cells

**Fig. 3:**
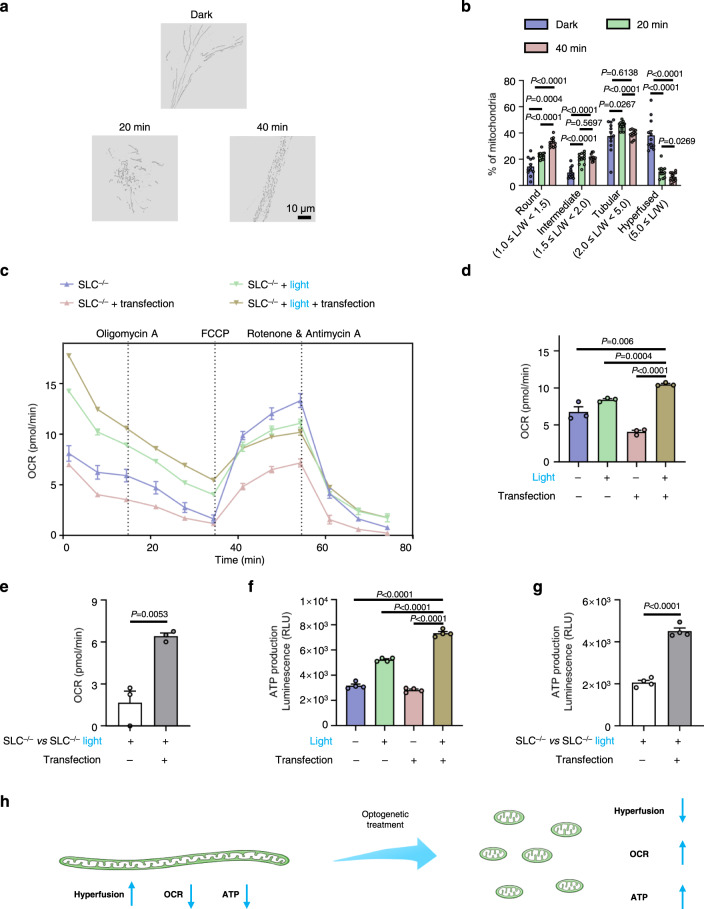
The optogenetic MLCs system reduces mitochondrial hyperfusion and restores mitochondrial function in SLC25A46^−/−^ cells. **a** Representative SIM images of mitochondria with different blue-light time (0, 20, and 40 min) in living SLC25A46^−/−^ HDFn cells expressing *LAMP–CRY2* and *TOM20–CIB–GFP*, and stained with MTR. **b** Quantitative analysis of mitochondrial morphology in SLC25A46^−/−^ HDFn cells after optogenetic treatments. “L/W” stands for dividing the length (L) by the width (W). *n* = 12 cells examined over 3 independent experiments. Data are presented as *M* ± *SEM* (**c**) The OCR curves of SLC25A46^−/−^ HDFn cells in real-time after different treatments (Data are presented as *M* ± *SEM* from 3 independent experiments.). OCR before the addition of oligomycin A indicates the basal respiration; OCR after the injection of FCCP denotes the maximal mitochondrial respiration capacity; and OCR after the injection of rotenone and antimycin A shows non-mitochondrial respiration. **d** The basal respiration of SLC25A46^−/−^ HDFn cells after different treatments (Data are presented as *M* ± *SEM* from 3 independent experiments). **e** The significance analysis of the basal respiration with optogenetic treatment (Data are presented as *M* ± *SEM* from 3 independent experiments). **f** The ATP production of SLC25A46^−/−^ HDFn cells after different treatments (Data are presented as *M* ± *SEM* from 4 independent experiments.). **g** The significance analysis of the ATP production with optogenetic treatment (Data are presented as *M* ± *SEM* from 4 independent experiments.). **h** Schematic representation of optogenetic treatment for SLC25A46^−/−^ HDFn cells. “SLC^−/−^ *vs* SLC^−/−^ light” refers to the value difference for SLC25A46^−/−^ HDFn cells before and after light stimulation. For (**b**) and (**d**–**g**), the statistical differences between the experimental groups were analyzed by double-tailed Student’s *t* test. When *P* < 0.05, it was considered to have statistical significance. Source data are provided as a Source Data file.

Deletion of SLC25A46, a member of the mitochondrial solute carrier family 25, reduces respiration and produces a hyperfused mitochondrial phenotype^21,22,38–40^. Indeed, the oxygen consumption rate (OCR) is lower in SLC25A46^−/−^ human dermal fibroblasts, neonatal (HDFn) cells compared with wild-type (Supplementary Fig. 25a), and more mitochondria appeared hyperfused (Supplementary Fig. 25b) under fluorescent imaging. To test whether the optogenetic MLCs system could restore mitochondrial function in the mutated cells, we transfected the plasmids into the SLC25A46^−/−^ HDFn cells. Under blue light illumination, we observed fragmentation of the hyperfused mitochondria induced by light-controlled MLCs (Fig. 3a, b, Supplementary Fig. 26, and Table 3). Morphology of mitochondrial cristae did not change after blue light exposure (Supplementary Fig. 27). The optogenetic MLCs system can thus reduce mitochondrial hyperfusion in SLC25A46^−/−^ HDFn cells.

We then examined mitochondrial bioenergy in SLC25A46^−/−^ HDFn cells after optogenetic treatment with light exposure for 20 min. The untreated control SLC25A46^−/−^ HDFn cells displayed several changes in the OCR curve (Fig. 3c), including an initial basal OCR level, attenuated ATP-linked respiration following the addition of oligomycin (i.e., the electron transport inhibitor), a rebound of the OCR peak after addition of FCCP (i.e., the electron chain transport accelerator), and a substantial drop after a mixture of rotenone (i.e., the complex I inhibitor) and antimycin A (i.e., the complex III inhibitor) was added to discontinue the mitochondrial respiratory chain. Seahorse experiments revealed that optogenetically treated cells improved the basal OCR level (Fig. 3c–e), thereby suggesting that the optogenetic treatment can enhance the mitochondrial OXPHOS pathway. Comparing to the wild-type SLC25A46^+/+^ HDFn cells, the optogenetic MLCs system restored respiration in SLC25A46^−/−^ HDFn cells to 80.23% (Fig. 3c and Supplementary Fig. 25a). Mitochondria are well-known cellular powerhouses, and enhanced cell metabolism with OCR level is expected to accompany increased ATP production^3,6^. To further describe that metabolic potential, we recorded the ATP production of SLC25A46^−/−^ HDFn cells treated in the same way. Compared to the control groups, ATP levels were enhanced in SLC25A46^−/−^ HDFn cells under optogenetic treatment (Fig. 3f, g). Taken together, the results indicate that the optogenetic MLCs system can partially restore the mitochondrial functions of SLC25A46^−/−^ HDFn cells and consequently improve OXPHOS and ATP levels (Fig. 3h).

## Discussion

We have developed a method to control mitochondrial fission via light-induced MLCs in multiple cell types, including HeLa cells, PC12 cells, and SLC25A46^−/−^ HDFn cells, and partially restored mitochondrial functions in SLC25A46^−/−^ HDFn cells. Compared to previously reported methods for mitochondrial fission, our optogenetic MLCs system possesses advantages (Supplementary Fig. 28). First, unlike chemical and biological methods, mitochondrial fission induced by the optogenetic tool is reversible. For the light-excited CRY2–CIB interaction, lysosomes and mitochondria can separate after withdrawing illumination, while mitochondrial fusion restores mitochondrial morphology. Secondly, the optogenetic method affords highly localized spatial control. Mitochondrial fission would increase in the area exposed to light, while other areas remain unchanged. Thirdly, chemically-induced mitochondrial fission often results in mitochondrial damage and cellular toxicity, whereas the optogenetic MLCs system mobilizes a cellular mechanism that protects mitochondria. Finally, biological methods require deletion of functional proteins, abolishing their other functions in cells, while the optogenetic method maintains the integrity of proteins. However, we recognize there are some minor issues for our optogenetic fission system require future developments, including the low penetrability of blue light, the inefficient transfection of two plasmids, and the homo-oligomerization of CRY2 on lysosomes.

The molecular mechanism of contact-induced mitochondrial fission was proposed whereby MLCs recruit both TBC1D15 and FIS1 proteins to indirectly achieve fission through RAB7 GTP hydrolysis^5,28^. This idea is supported by our result that RAB7 knock-down reduced the light-induced mitochondrial fission (Supplementary Fig. 29). Organelles play multiple roles in cells, which are regulated by their interaction. The effective collision of organelles for initiating protein interactions would be a rate-limiting step for the occurrence. Under blue light illumination, the photo-activated protein system CRY2-CIB rapidly brings mitochondria and lysosomes together to form MLCs, bypassing the slow, endogenous MLCs process. Meanwhile the light-induced MLC by the CRY2-CIB interaction is different with the endogenous MLC, which is formed by the TBC1D15-RAB7 interface for fission. Therefore, with the link of CRY2-CIB, the endogenous fission mechanism remains. Moreover, as shown by Wong et al.^28^, there is a 10-nm gap between two membranes of contacted lysosomes and mitochondria, which leaves some space for protein dynamics, such as the untethering of lysosomes by RAB7 GTP hydrolysis. Our optogenetic system provides an efficient tool to continuously initiate the TBC1D15-RAB7 mechanism for mitochondrial fission.

Meanwhile, other mechanisms for mitochondrial fission have also been proposed recently^41–46^. Endoplasmic reticulum (ER) has been reported to be involved in mitochondrial fission induced by MLCs, and it is believed that mitochondrial fission occurs at three-way organelle junctions between mitochondria, ER tubules, and lysosomes^28,47^. For the ER-lysosome co-induced fission, an ER-lysosome tethering protein ORP1L (the cholesterol sensor oxysterol-binding protein-related protein 1L) was required, and VAPB-ORP1L-RAB7 (VAPB, vesicle-associated membrane protein-associated protein B) mediated ER-lysosomes contact recruiting lysosomes to the site of mitochondrial fission^41^. RAB7 plays a critical role in regulating both the lysosome-mediated fission reported by Wong et al.^28^ and the ER-lysosome co-induction model suggested by Boutry et al.^41^. Furthermore, a recent result reported by Kleele et al.^42^ revealed the two lysosome-dependent mitochondrial fission events, center and peripheral fission, which can occur simultaneously. During the optogenetic MLCs, it is possible that the VAPB-ORP1L-RAB7 model also takes part to induce mitochondrial fission. Again, the detailed mechanisms for the mitochondrial fission under various conditions need to be studied further.

Mitochondrial dynamics is closely involved in metabolism, and mitochondrial fission can either benefit or impair mitochondria and cellular metabolism^48^. With optogenetically induced mitochondrial fission, the OCR level of HeLa cells decreased due to the imbalance of mitochondrial dynamics (Supplementary Fig. 30). Meanwhile, blue light has been reported to impact mitochondrial functions by altering mitochondrial fusion and fission imbalance or upregulating complexes III, IV and V^49–53^. However, the significance analysis (Fig. 3e, g) showed that optogenetic treatment is the major factor for the changes in mitochondrial functions. SLC25A46^−/−^ cells show a hyperfused mitochondria phenotype. Balancing mitochondrial dynamics, however, can restore functional integrity, and gene therapy has been used to combat this mitochondrial disorder in mice^54^. In our work, the mitochondrial functions of SLC25A46^−/−^ cells were partially restored by mitochondrial fission induced by the optogenetic MLCs system, which affords a potential method for treating this type of mitochondrial disorder.

In summary, we have developed an optogenetic strategy for controlling mitochondrial fission in living cells with reversibility and spatiotemporal controllability. The method can be used to counter hyperfused mitochondria and restore mitochondrial biological functions in mutated cells, and the optogenetic MLCs system shows promise in studying mitochondrial fission and treating mitochondrial diseases.

## Methods

### Materials

MitoTracker^TM^ Green FM (MTG, #M7514), MitoTracker^TM^ Red FM (MTR, #M22425), MitoTracker^TM^ Deep Red FM (MTDR, #M22426) and LysoTracker^TM^ Red DND–99 (LTR, #L7528) were purchased from Invitrogen (Thermo Fisher Scientific, USA). Penicillin–streptomycin (#15140163, 10,000 units/mL), fetal bovine serum (FBS, #26140079), and DMEM (#11965092) were all purchased from Gibco (Thermo Fisher Scientific, USA). Phosphate-buffered saline (PBS, #SH30256.01) was purchased from Hyclone (GE Healthcare Life Sciences, USA). *LAMP–mCherry–CRY2* (Addgene plasmid, #102249), *TOM20–CIB–GFP* (Addgene plasmid, #117242), and *CRY2olig-mCherry* (Addgene plasmid, #60032) were gifts from Bianxiao Cui, Robert Hughes, and Chandra Tucker, respectively. TurboFect^TM^ Transfection Reagent (#R0532) was purchased from Thermo Scientific (Thermo Fisher Scientific, USA). To construct the plasmid *LAMP–CRY2*, QuiKChange II XL Site–Directed Mutagenesis Kit (#200521, Agilent) was used to mutate mCherry in the plasmid *LAMP–mCherry–CRY2* according to the manufacturer’s protocol. The reverse primer tcgcccttgctcactgcggtggcgaccggtgg and the forward primer ccaccggtcgccaccgcagtgagcaagggcga were purchased from Integrated DNA technologies. RAB7 siRNA (h, sc-29460) and the antibody RAB7 (mouse, B-3, #sc-376362, 1:1000) were purchased from Santa Cruz Biotechnology Inc (USA). *CRY2PHR-mCherry* (CRY2PHR, residues 1–498 of cryptochrome 2; Addgene plasmid, #26866) and *CIB-GFP-CAAX* (CAAX: cysteine, two aliphatic, and variable (X) C-terminal amino acids; Addgene plasmid, #26867) were gifts from Dr. Chandra Tucker at the University of Colorado Denver. *CRY2PHR-mCherry-Raf1* (Raf1, Raf-1 proto-oncogene serine/threonine kinase) was constructed through overlap PCR (polymerase chain reaction) by inserting Raf1 gene into the backbone of *CRY2PHR-mCherry* with sense primer (ctgtacaagtccggactcagatctcgagtgatggagcacatacagggagcttggaagacg) and anti-sense primer (acgggccctctagactcgagcggccgcttagaagacaggcagcctcggggacgtggtcag). For generating *TOM20-CRY2-GFP* plasmid, we used a previously developed plasmid *TrkICD-CRY2-GFP* and amplified the entire backbone including only *CRY2-GFP* and then introduced TOM20 upstream of this using Gibson assembly. *TOM20-CRY2-GFP* plasmid was amplified again excluding CRY2 this time and we introduced SspB in place using Gibson assembly. For generating *LAMP-mCherry-CIB* and *LAMP-mCherry-iLID*, we used the plasmid *LAMP-mCherry-CRY2* used in this study. Briefly, we amplified the backbone with only LAMP and incorporated *mCherry-iLID* or *mCherry-CIB* downstream using Gibson assembly.

### Cell culture and transfection

The HeLa cell line (a gift from Dr. Carolyn M. Price, University of Cincinnati), PC12 cell line (a gift from Prof. Tobias Meyer, Stanford University) and HDFn cell lines (wild-type and SLC25A46^−/−^) were cultured in DMEM containing 10% FBS and 100 units/mL of penicillin–streptomycin in a 5% CO_2_ cell incubator (Thermo Fisher Scientific, USA) with 100% humidity at 37 °C. Transfection was performed using TurboFect^TM^ Transfection Reagent according to the manufacturer’s protocol. *LAMP–mCherry–CRY2* and *TOM20–CIB–GFP* (ratio 1:1) were mixed with 7.2 μL TurboFect in 240 μL DMEM medium (without FBS) for 20 min. The DNA/Turbofect mixtures were added to the cell cultures drop-wise and incubated for 6 h before replenishment with complete culture medium. Geneticin^TM^ selective antibiotic (#10131035, Gibco, Thermo Fisher Scientific, USA) at 500 ng/mL was used to select the expressed cells.

BHK-21 cells (baby hamster kidney fibroblast cells, a gift from Dr. Xiaolin Nan, Oregon Health & Science University) were cultured in DMEM supplemented with 10% FBS and penicillin–streptomycin. Before transfection, cells were plated on poly-L-lysine-coated glass coverslip (VWR) with a home-made polydimethylsiloxane (PDMS, Ellsworth) chamber. For co-transfection, a mixture of *CRY2PHR–mCherry–Raf1* (2 μg) and *CIB–GFP–CAAX* (1.1 μg) was mixed with 6 μL Turbofect in 386.5 μL DMEM. All mixtures were incubated at room temperature for 20 min. The DNA/Turbofect mixtures were added to the cell cultures drop-wise and incubated for 3 h before replenishment with complete culture medium.

### Confocal fluorescence imaging

Fluorescence imaging of the transfected BHK21 cells was carried out using a confocal microscope (Zeiss LSM 700). GFP fluorescence was excited by a 488-nm laser beam; mCherry fluorescence was excited by a 555-nm laser beam. Excitation beams were focused via a 40× oil objective (Plan-Neofluar NA 1.30). Ten pulses of 488-nm and 555-nm excitation light (2-s interval) were applied for each membrane recruitment experiment. CRY2–CIB binding induced by 488-nm light was monitored by membrane recruitment of *CRY2–mCherry–Raf1* to the plasma membrane-anchored *CIB–GFP–CAAX*. The powers after the objective for 488-nm and 555-nm laser beam were approximately 40 µW and 75 µW, respectively.

### Structured illumination microscopy imaging

All SIM images were performed with a Nikon structured illumination microscopy (N-SIM, version AR5.11.00 64 bit, Tokyo, Japan), a 3D-SIM equipped with an Apochromat 100×/1.49 numerical aperture oil-immersion objective lens and solid-state lasers (488 nm, 561 nm, 640 nm, the output powers at the fiber end: 15 mW). Images were captured using Nikon NIS-Elements 512 × 512 using Z-stacks with a step size of 0.2 μm and the raw images were reconstructed and processed with NIS-Elements AR Analysis (version AR5.11.00 64 bit). The green channel images with emission bandwidth at 500–550 nm were excited by a 488 nm laser for MTG and GFP. The red channel images with emission bandwidth at 570–640 nm were excited by a 561 nm laser for MTR, LTR, and mCherry. The deep red channel images with emission bandwidth at 660–735 nm were excited by a 640 nm laser for MTDR. Cells were seeded on glass-bottomed culture dishes (MatTek; P35G-1.5-14-C) for 24 h to adhere. Staining with commercial dyes were performed for 30 min. Before imaging, cells were washed with PBS 3 times. For illumination, a homemade blue light emitting diode (LED) array was used with 300 μW/cm^2^. The imaging data analysis was performed with ImageJ.

### Mitochondrial morphology quantification

Mitochondria were labelled by the fluorescence protein GFP or MitoTracker dyes (MTG, MTR, or MTDR). After acquiring from SIM, fluorescent images containing bright mitochondria in individual cells were imported into ImageJ (NIH). The image type was changed to 8-bit with an adjusted threshold. After selecting area, shape descriptors, and limit to the threshold using the “set measurements” function, results can be obtained through the “analyze particles” function. The data in the “AR” column is the ratio of “L/W”.

To capture mitochondrial fission caused by light-induced MLCs in real time, we performed the experiment at room temperature to decelerate cellular enzyme activity. After optogenetically induced fission, the mitochondria were divided into four groups for quantitative analysis^55,56^. *TOM20–CIB–GFP* was on the mitochondrial membrane, and its GFP fluorescence indicates the profile of mitochondrion, not a full view. To capture the panorama of mitochondria, *TOM20–CIB–GFP/LAMP–CRY2* (i.e., without mCherry) and available mitochondria–targeting dye MTR (or MTG, MTDR) were used in the experiment. The fluorescence in the images was analyzed by ImageJ, and the difference between the images from the two fluorescence tags appears in Supplementary Fig. 31. By using those dyes to capture the panorama of a mitochondrion, we obtained more reliable results for quantitative analysis.

### Oxygen consumption rate assay

To examine the mitochondrial OXPHOS function under blue light exposure, the oxygen consumption rate (OCR) of SLC25A46^−/−^ cells expressing *LAMP–mCherry–CRY2* and *TOM20–CIB–GFP* was measured using a Seahorse XFe-96 Analyzer (Agilent Technologies, USA) in real time. SLC25A46^−/−^ cells (two groups, one for illumination and one for dark) and SLC25A46^–/–^ cells expressing *LAMP–mCherry–CRY2* and *TOM20–CIB–GFP* (two groups, one for illumination and one for dark) were seeded in a XFe-96 cell culture plate (Agilent Technologies, USA) at 0.8 × 10^4^ cells/well in DMEM with 10% FBS. After 48 h, the medium was removed and replaced with the warmed unbuffered “Seahorse medium” (XF DMEM with 1 mM sodium pyruvate, 10 mM glucose and 2 mM L-glutamine) at pH 7.4. Before the measurement, one group of SLC25A46^−/−^ cells and one group of SLC25A46^−/−^ cells expressing plasmids were exposed to blue light (300 μW/cm^2^) for 20 min. Then the OCRs of the cells were assessed using the XF Cell Mito Stress Test Kit (Agilent Technologies, USA) according to the manufacturer’s instruction. Where indicated, the cells were treated with 1 μM oligomycin A (the electron transport inhibitor), 2 μM FCCP (the electron chain transport (ETC) accelerator), and 500 nM rotenone (the complex I inhibitor) with 1 μM antimycin A (the complex III inhibitor, all are from Agilent Technologies).

### ATP quantification assay

For the ATP quantification assay, SLC25A46^−/−^ cells (two groups, one for illumination and one for dark) and SLC25A46^−/−^ cells expressing *LAMP–mCherry–CRY2* and *TOM20–CIB–GFP* (two groups, one for illumination and one for dark) were seeded into a 6-well plate (Corning, USA). After 24 h, one group of SLC25A46^−/−^ cells and one group of SLC25A46^−/−^ cells expressing plasmids were exposed to blue light (300 μW/cm^2^) for 20 min. Then the cells were harvested and treated according to the manufacturer’s protocol of the ATP Bioluminescence Assay Kit CLS II (catalog no. 11699695001, Roche). The supernatant of the treated cells and the ATP standard were transferred into a white walled nontransparent bottomed 96-well plate (Corning, USA). The luminescence was read by a microplate reader (BioTek Instruments, Inc., USA).

### Cell viability test

Cell Counting Kit-8 (CCK-8, Dojindo Molecular Technologies, Inc., Japan) was used to determine the photocytotoxicity of blue-light. The cells (HeLa cells, PC12 cells and SLC25A46^−/−^ cells) were seeded into two groups in a 96-well plate with 1 × 10^4^ cells/well. After 24 h to adhere, the light group was exposed to blue-light (300 μW/cm^2^) for 60 min, while the dark group was covered with tin foil sheet and left unchanged. Then 10 μL of CCK-8 solution was added to each well, the culture plate was incubated for 1 h. Absorbance at 490 nm was determined with the Synergy Mx microplate reader (BioTek Instruments, Inc., USA).

### Data analysis

Statistical significance of data was evaluated using Student’s *t* test or one-way analysis of variance (ANOVA) with Tukey’s post hoc test (for multiple datasets). Data were presented as *M* ± *SEM*. Statistics and graphing were performed using Prism 8 (GraphPad) or Excel (Microsoft). The mitochondrial morphology was analyzed by ImageJ-win64 (NIH Image). All images were assembled using PowerPoint 2016 software (Microsoft).

### Statistics and reproducibility

Each experiment was repeated at least three times independently with similar results. All images shown are representative results from biological replicates.

### Reporting summary

Further information on research design is available in the Nature Research Reporting Summary linked to this article.

## Supplementary information

Supplementary figures and tables

Editorial Assessment Report

Description of additional Supplementary Information

Movie 1

Movie 2

Movie 3

Reporting Summary

## Data Availability

All data supporting the findings of this study are available either in the article and/or its Supplementary Information files. Source data are provided with this paper.

## Source data

Source Data File
