# Supplementary material for: Light-activated mitochondrial fission through optogenetic control of mitochondria-lysosome contacts
Source: Nat Commun. 2022 Jul 25;13:4303. doi: 10.1038/s41467-022-31970-5 (PMC9314359; doi:10.1038/s41467-022-31970-5)
Supplement: Supplementary file 1 — Supplementary figures and tables [file 41467_2022_31970_MOESM1_ESM.pdf]

Supplementary Material

Light-activated mitochondrial fission through optogenetic control of mitochondria-lysosome contacts

Kangqiang Qiu[1,†], Weiwei Zou[2,†], Hongbao Fang[1], Mingang Hao[1], Kritika Mehta[3], Zhiqi Tian[4], Jun-Lin Guan[1,*], Kai Zhang[3,*], Taosheng Huang[2,5,*], Jiajie Diao[1,*]

1 Department of Cancer Biology, University of Cincinnati College of Medicine, Cincinnati, OH 45267, USA.

2 Division of Human Genetics, Cincinnati Children's Hospital Medical Center, Cincinnati, OH 45229, USA.

3 Department of Biochemistry, School of Molecular and Cellular Biology, University of Illinois at Urbana-Champaign, Urbana, IL 61801, USA.

4 Department of Molecular Genetics, Biochemistry, and Microbiology, University of Cincinnati College of Medicine, Cincinnati, OH 45267, USA.

5 Department of Pediatrics, University at Buffalo, 1001 Main Street, Buffalo, NY 14203, USA.

† These authors contributed equally to this work.

* Corresponding author. Email: guanjl@ucmail.uc.edu (J.L.G.), kaizkaiz@illinois.edu (K.Z.), thuang29@buffalo.edu (T.H.), jiajie.diao@uc.edu (J.D.).

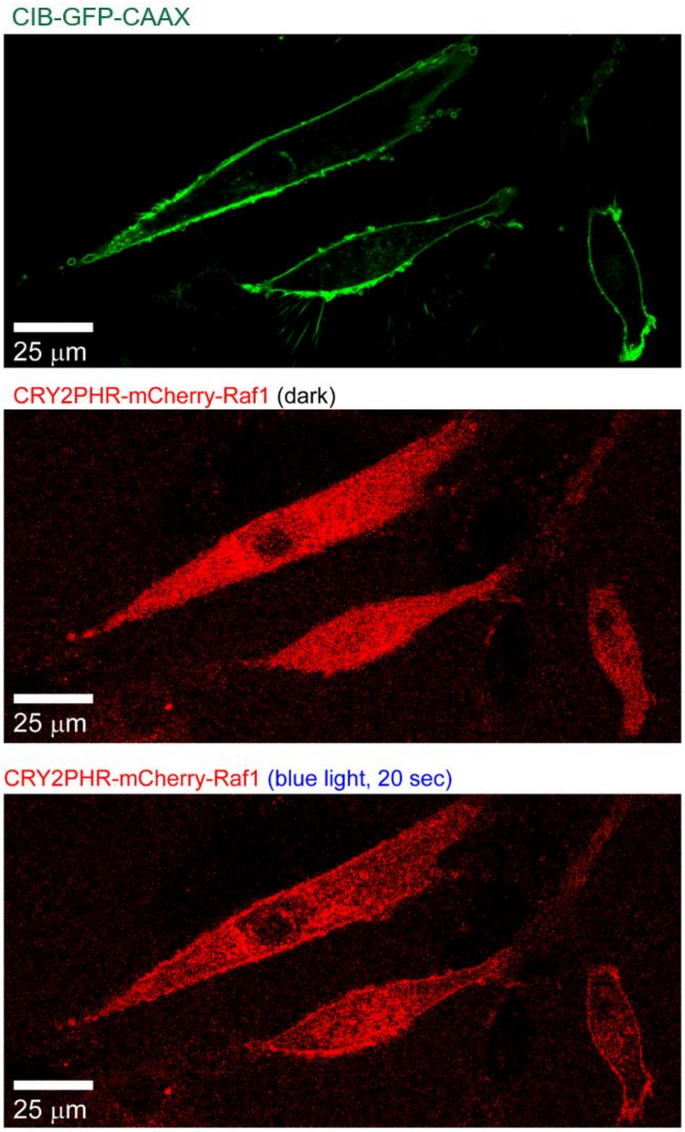

Supplementary Fig. 1 Blue light-induced association between CIB and CRY2PHR fusion proteins. BHK21 cells were co-transfected with *CIB-GFP-CAAX* and *CRY2PHR-mCherry-Raf1* before confocal fluorescence imaging. CIB was anchored to the plasma membrane via a CAAX domain (green) and CRY2 was fused to *mCherry-Raf1* (red). In the dark, *CRY2-mCherry-Raf1* is diffusive in the cytoplasm. Upon blue light stimulation (2 sec/frame, 10 frames), CIB-CRY2PHR association recruits the cytosolic CRY2PHR to the plasma membrane, as evidenced by the enhanced ratio of membrane to cytoplasmic mCherry fluorescence. Also see Video 1 for the time-stamp images.

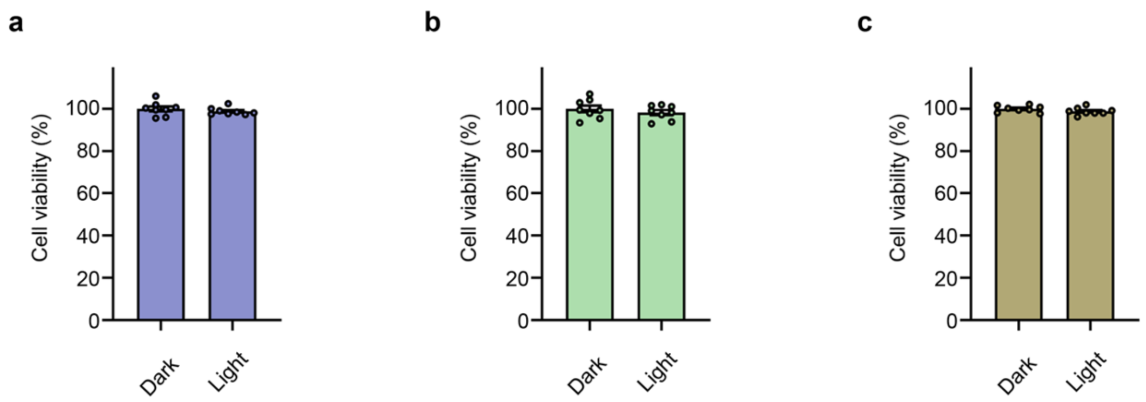

Supplementary Fig. 2 The viability of (**a**) HeLa cells, (**b**) PC12 cells, and (**c**) SLC25A46$^{-/-}$ HDFn cells with/without blue-light exposure for 60 min. The corresponding histograms (**a**, **b** and **c**) of the determined cell viability from n = 8 independent experiments. Data are presented as $M \pm SEM$. Source data are provided as a Source Data file.

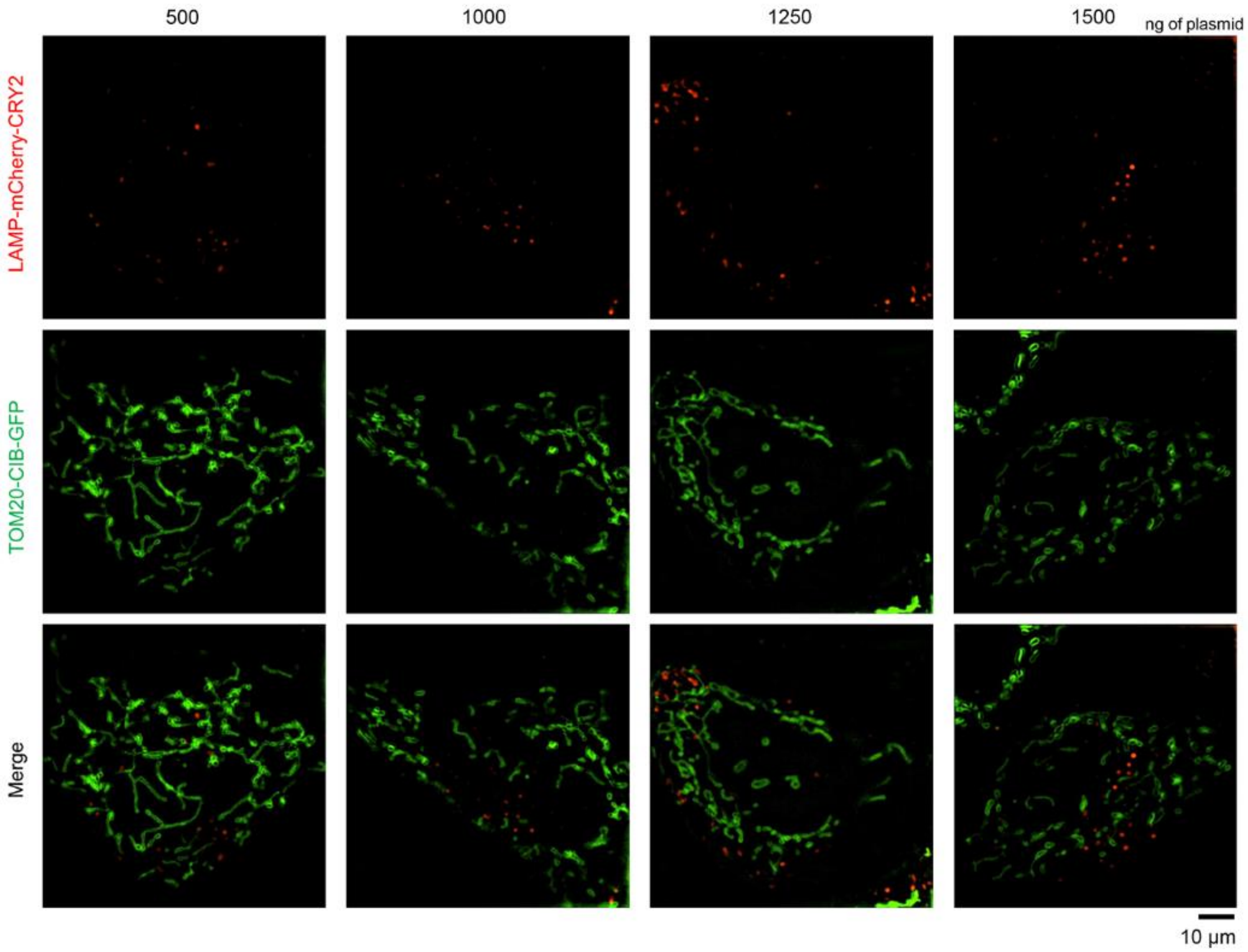

Supplementary Fig. 3 The SIM images of lysosomes (red) and mitochondria (green) in living HeLa cells expressing different concentrations of *LAMP–mCherry–CRY2* and *TOM20–CIB–GFP*. The concentration is for each plasmid. All images shared the same scale bar.

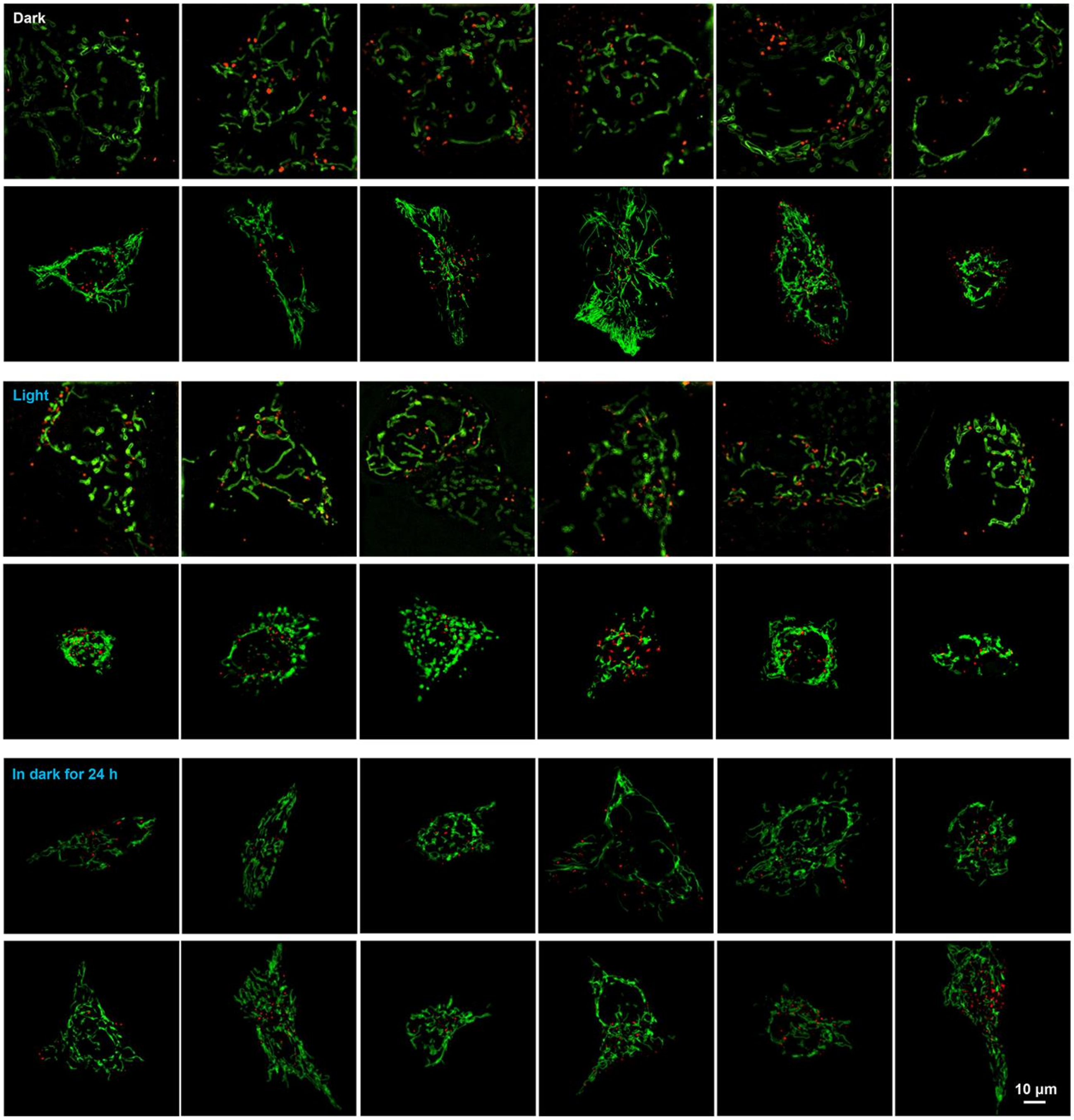

Supplementary Fig. 4 The data set for Fig. 1e and 1f. The SIM images of HeLa cells expressing *LAMP–mCherry–CRY2* and *TOM20–CIB–GFP* without or with blue light exposure, or after light exposure and then in dark for 24 h. All images shared the same scale bar.

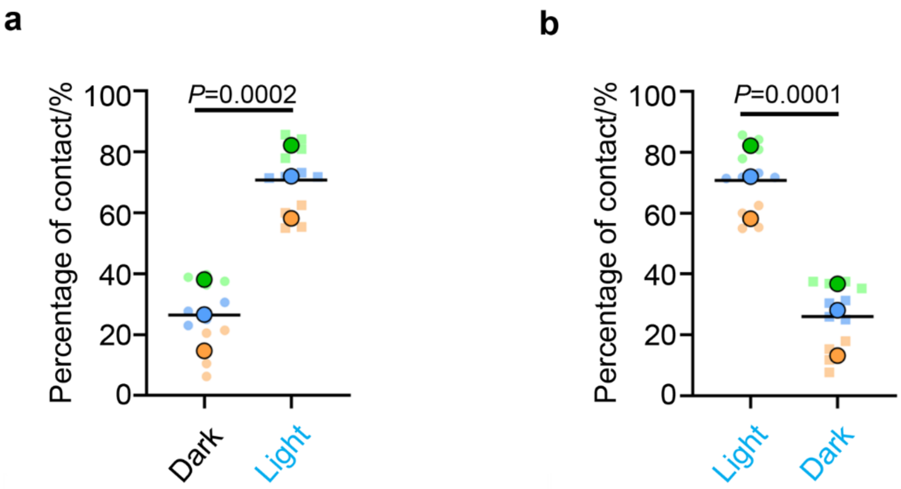

Supplementary Fig. 5 The superplots of the optogenetic inducing MLCs. (**a**) The percentage of contact before and after blue light exposure; (**b**) The percentage of contact after light exposure and then in dark for 24 h. n = 12 cells examined over 3 independent experiments. The statistical differences between the experimental groups were analyzed by double-tailed Student's t test. When $P < 0.05$, it was considered to have statistical significance. Source data are provided as a Source Data file.

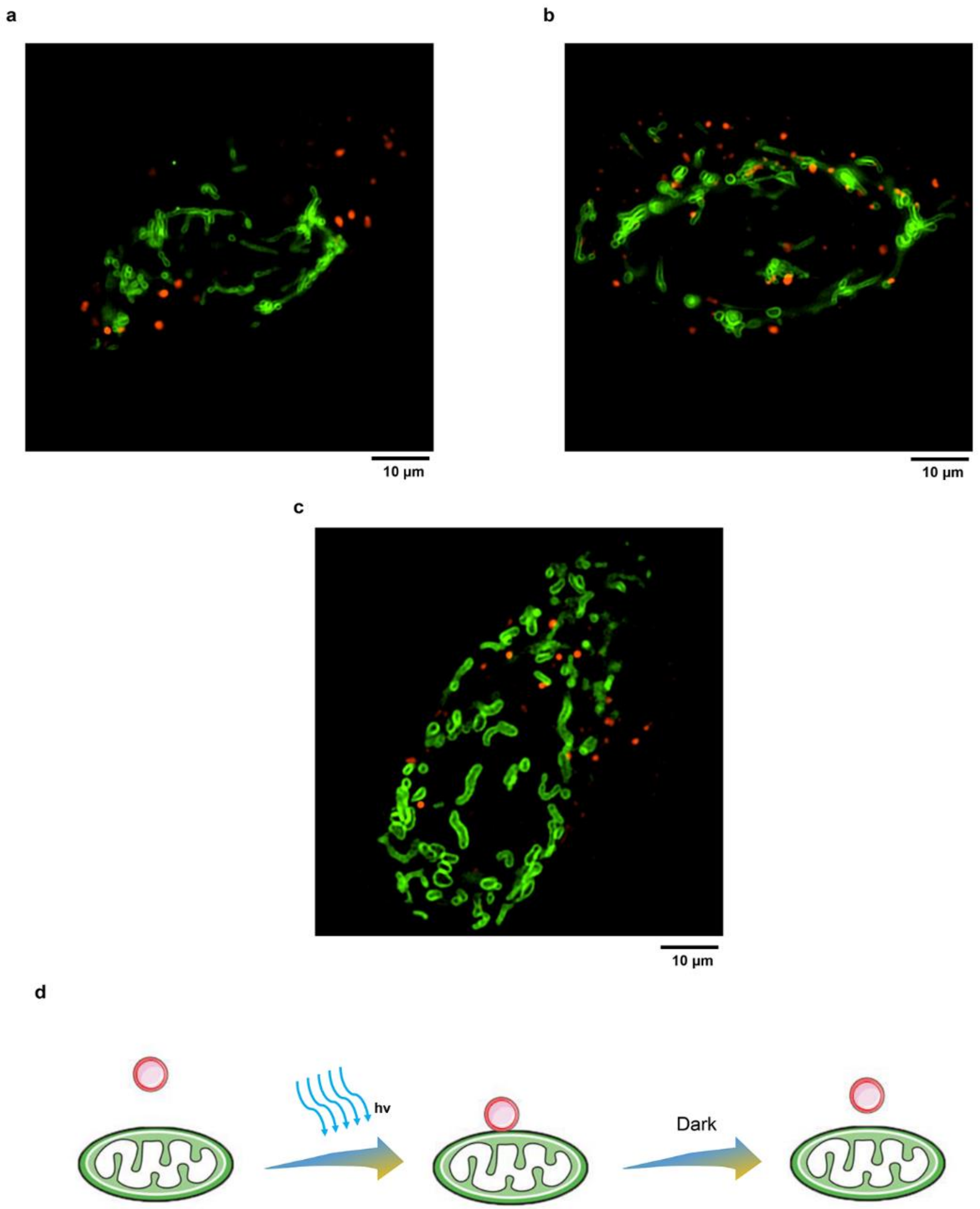

Supplementary Fig. 6 The reversibility of light-induced MLCs. (**a-c**) The SIM images of HeLa cells expressing *LAMP–mCherry–CRY2* and *TOM20–CIB–GFP*. (**a**) Before blue-light exposure; (**b**) After 20 min blue-light exposure; (**c**) After 20 min light illumination and transfer to dark for 20 min. (**d**) Schematic of the reversibility of light-induced MLCs.

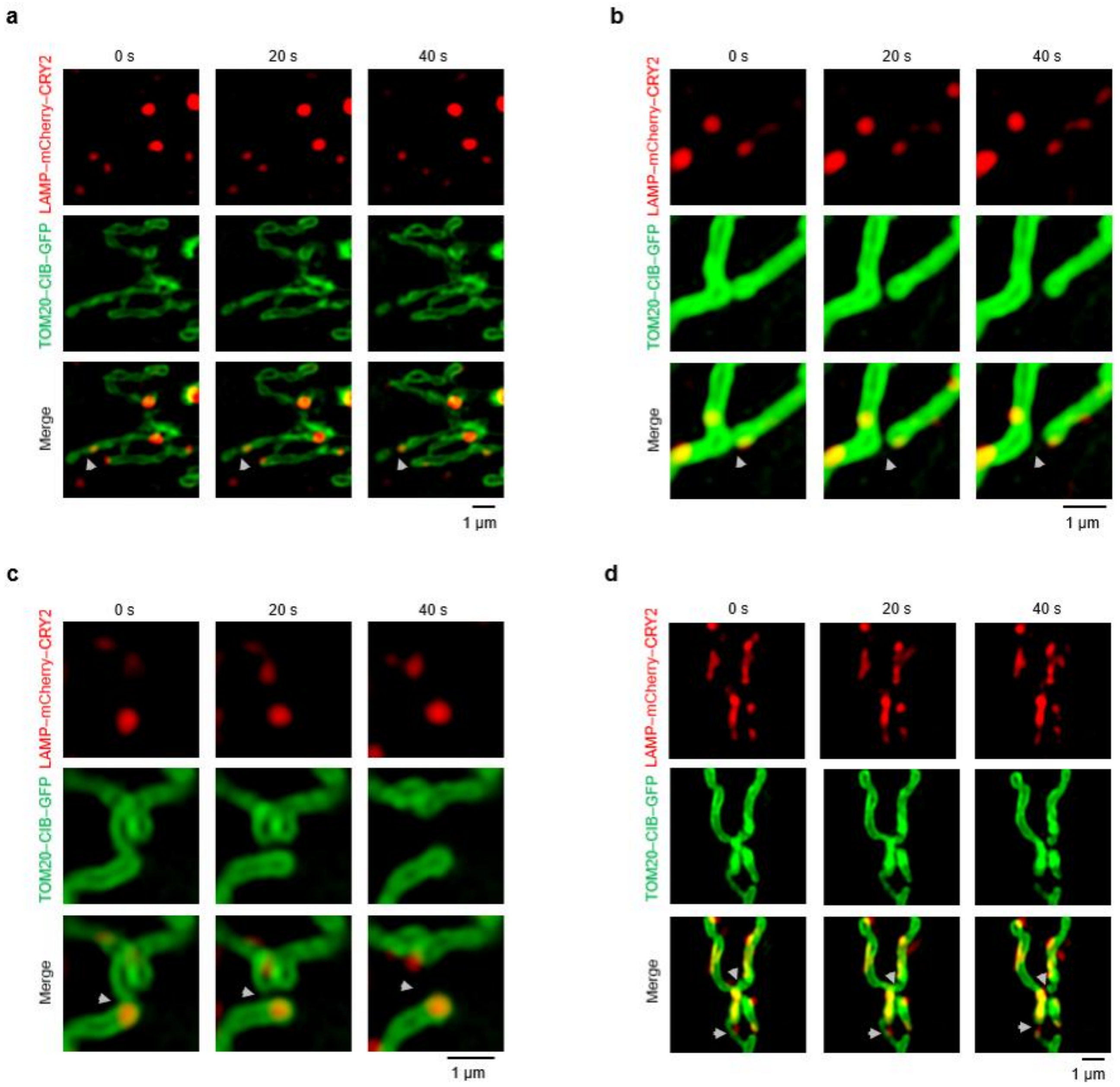

Supplementary Fig. 7 (**a–d**) Representative time-lapse images of lysosomes contacting mitochondria at site of mitochondrial division during mitochondrial fission in living HeLa cells expressing *LAMP–mCherry–CRY2* (lysosomes) and *TOM20–CIB–GFP* (mitochondria) under blue-light illumination. All images shared the same scale bar.

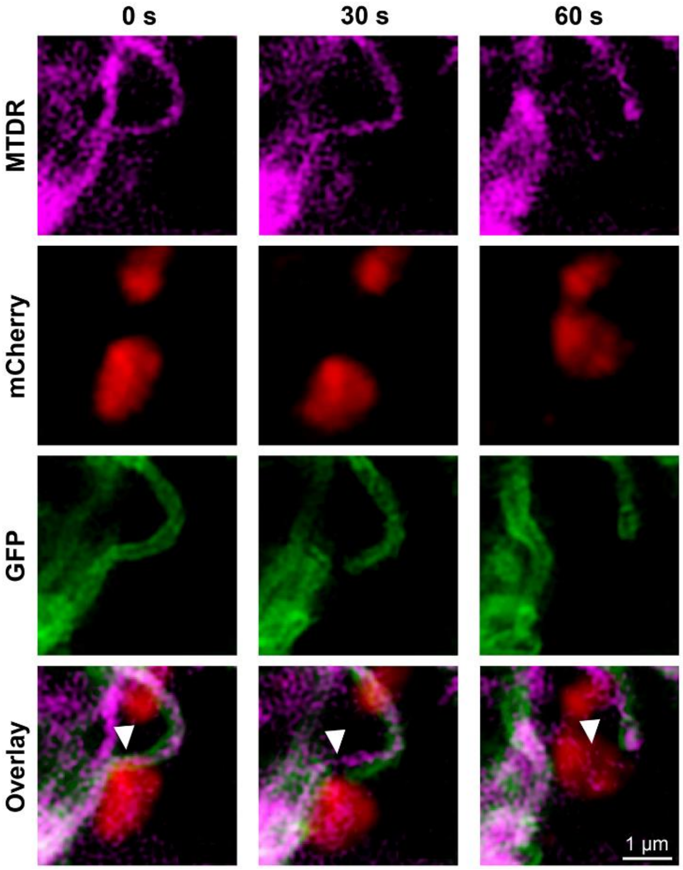

Supplementary Fig. 8 The real-time SIM images of HeLa cells expressing *LAMP-mCherry-CRY2* and *TOM20-CIB-GFP*, and staining MTDR under blue light exposure. White arrowheads mark the site of mitochondrial division. All images shared the same scale bar.

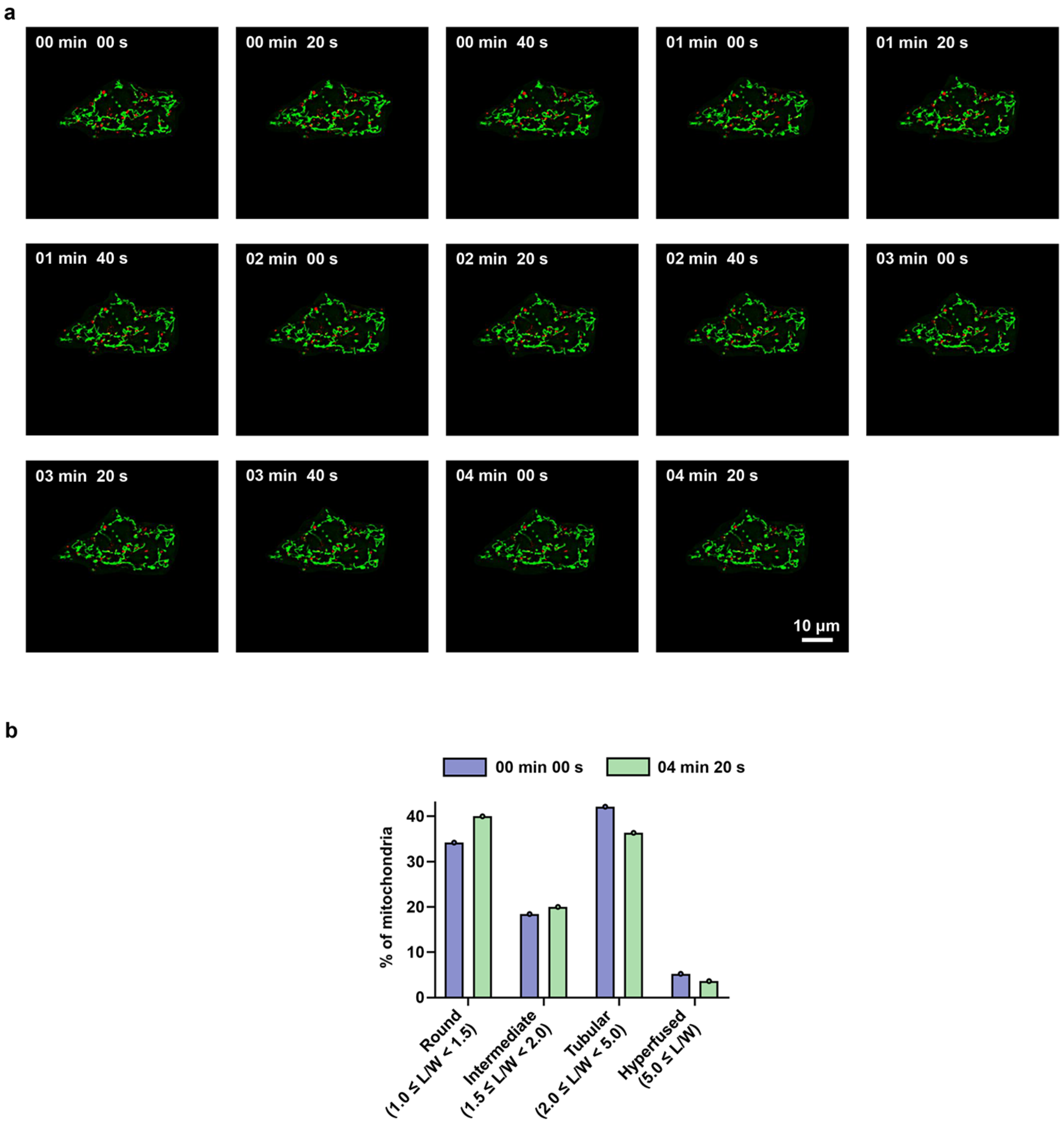

Supplementary Fig. 9 The real-time tracking of the same HeLa cell expressing *LAMP–mCherry–CRY2* and *TOM20–CIB–GFP*. (**a**) The time-lapse SIM images under blue light exposure. All images shared the same scale bar. (**b**) Quantitative analysis of mitochondrial morphology for HeLa cell before and after blue light exposure. Source data are provided as a Source Data file.

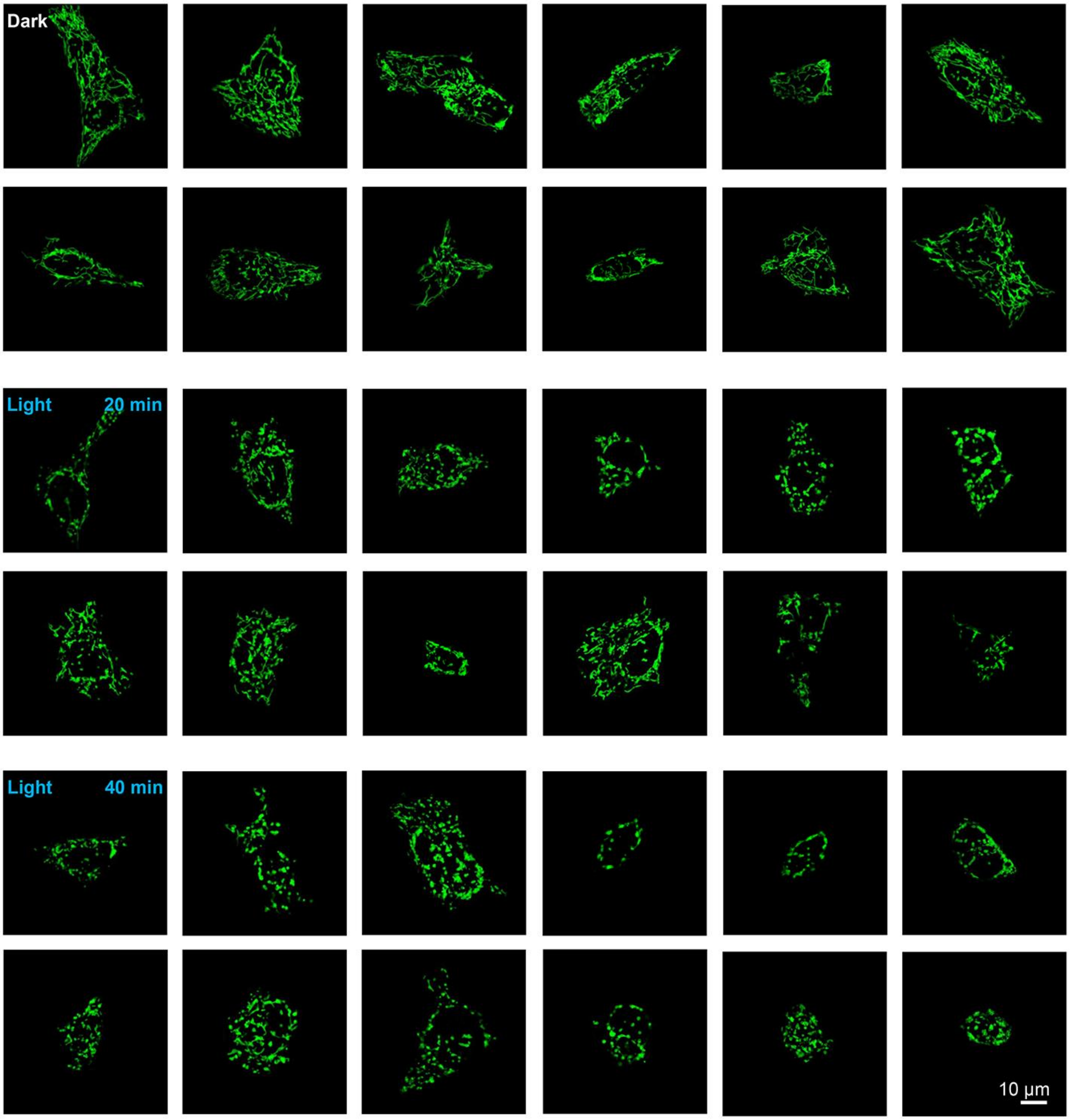

Supplementary Fig. 10 The data set for Fig. 2e. The SIM images of mitochondria in living HeLa cells expressing *LAMP–mCherry–CRY2* and *TOM20–CIB–GFP* under blue-light illumination for different times. All images shared the same scale bar.

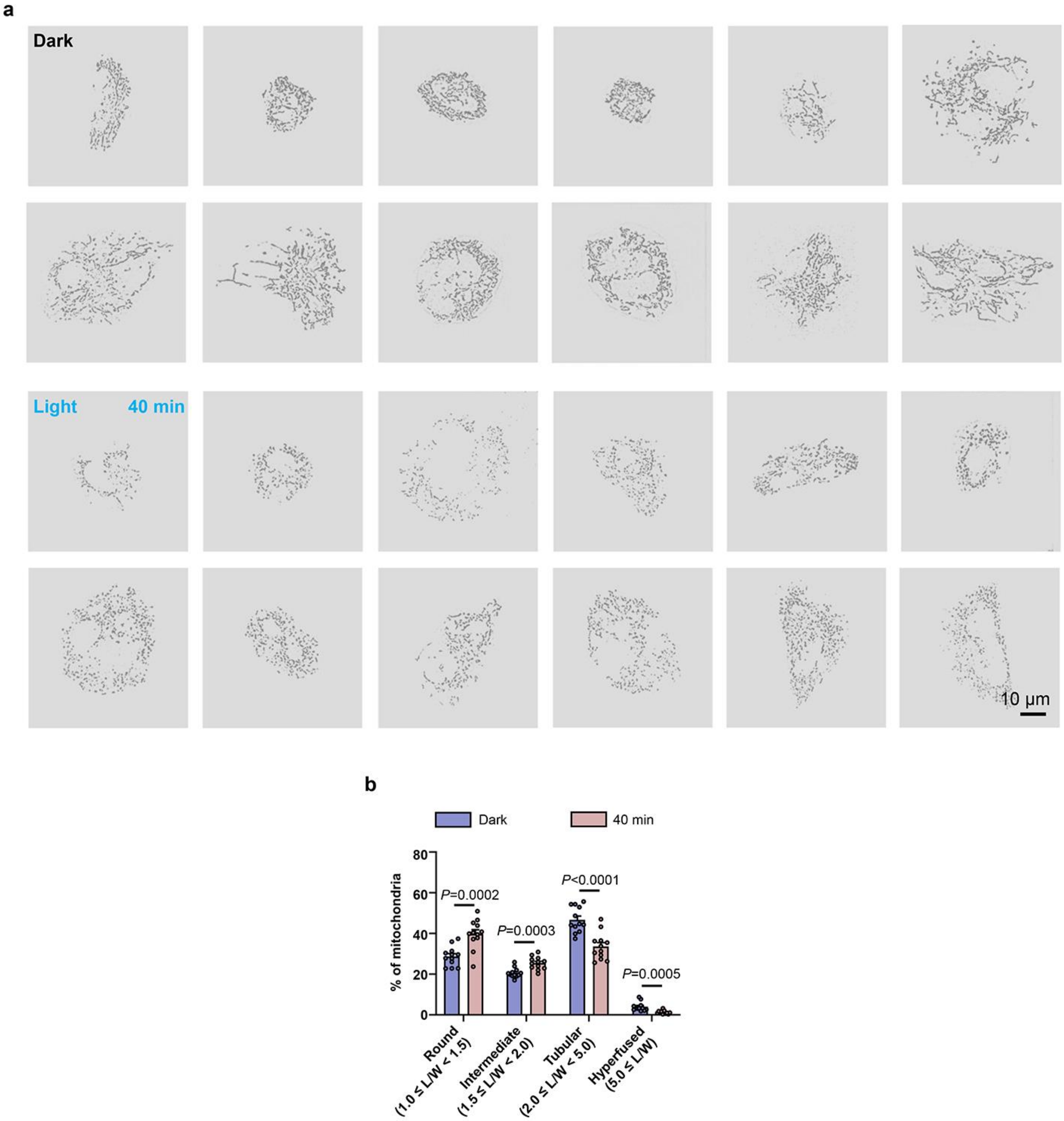

Supplementary Fig. 11 (**a**) The SIM images of mitochondria in living HeLa cells expressing *LAMP–CRY2* and *TOM20–CIB–GFP*, and stained with MTR under blue-light illumination for different times. All images shared the same scale bar. (**b**) Quantitative analysis of mitochondrial morphology of (a). n = 12 cells examined over 3 independent experiments. Data are presented as $M \pm SEM$. The statistical differences between the experimental groups were analyzed by double-tailed Student's t test. When $P < 0.05$, it was considered to have statistical significance. Source data are provided as a Source Data file.

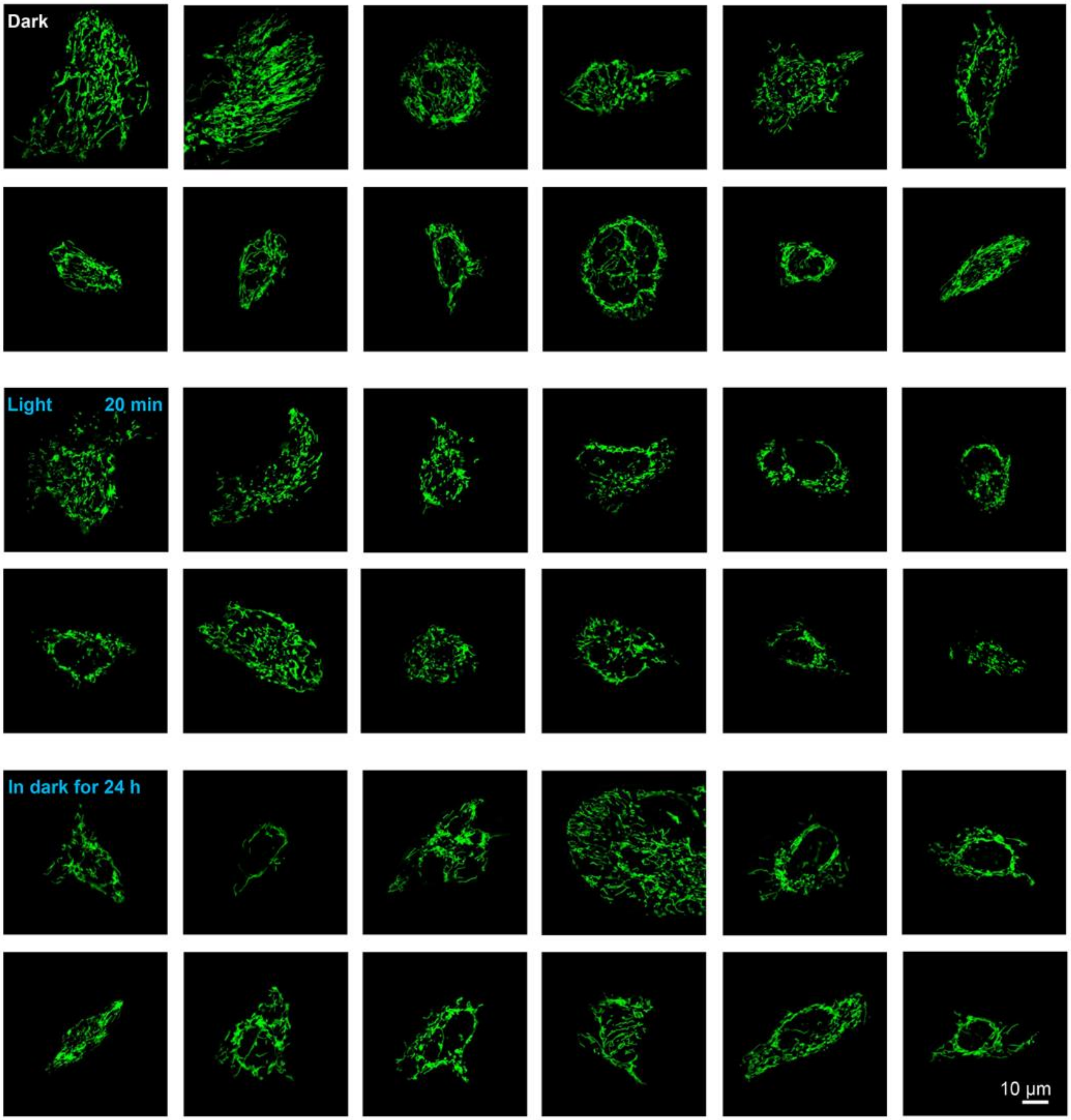

Supplementary Fig. 12 The data set for Fig. 2g. The SIM images of mitochondrial morphology for the reversibility of mitochondrial fission induced by optogenetic MLCs system in living HeLa cells expressing *LAMP–mCherry–CRY2* and *TOM20–CIB–GFP*, without or with blue light exposure, or after light exposure and then in dark for 24 h. All images shared the same scale bar.

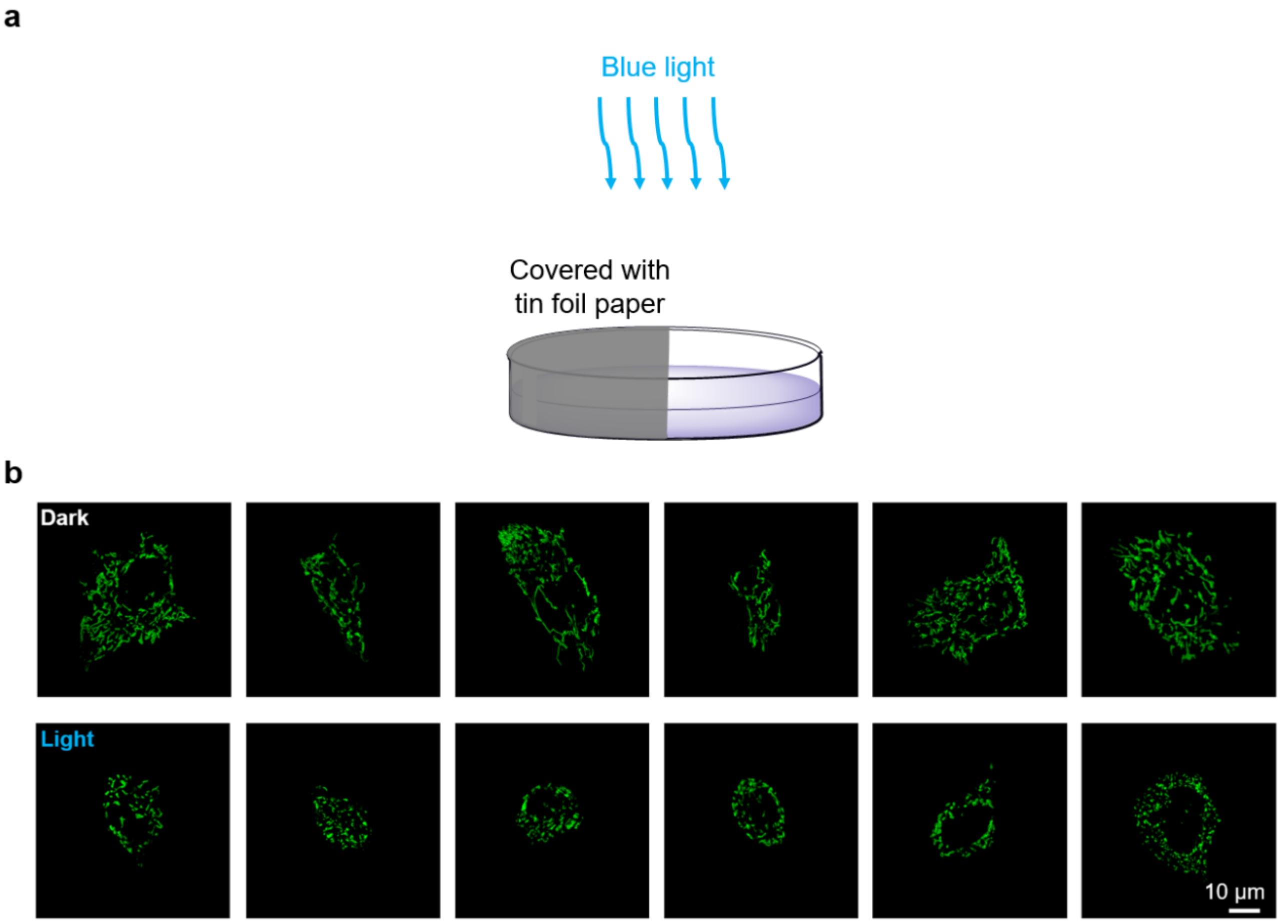

Supplementary Fig. 13 (**a**) Schematic representation of the spatial control experiment, where half of the culture dish was covered with tin foil paper to darken that portion of the dish. (**b**) The SIM images of mitochondria in HeLa cells expressing *LAMP–mCherry–CRY2* and *TOM20–CIB–GFP* by spatial control with or without light exposure for 20 min. All images shared the same scale bar.

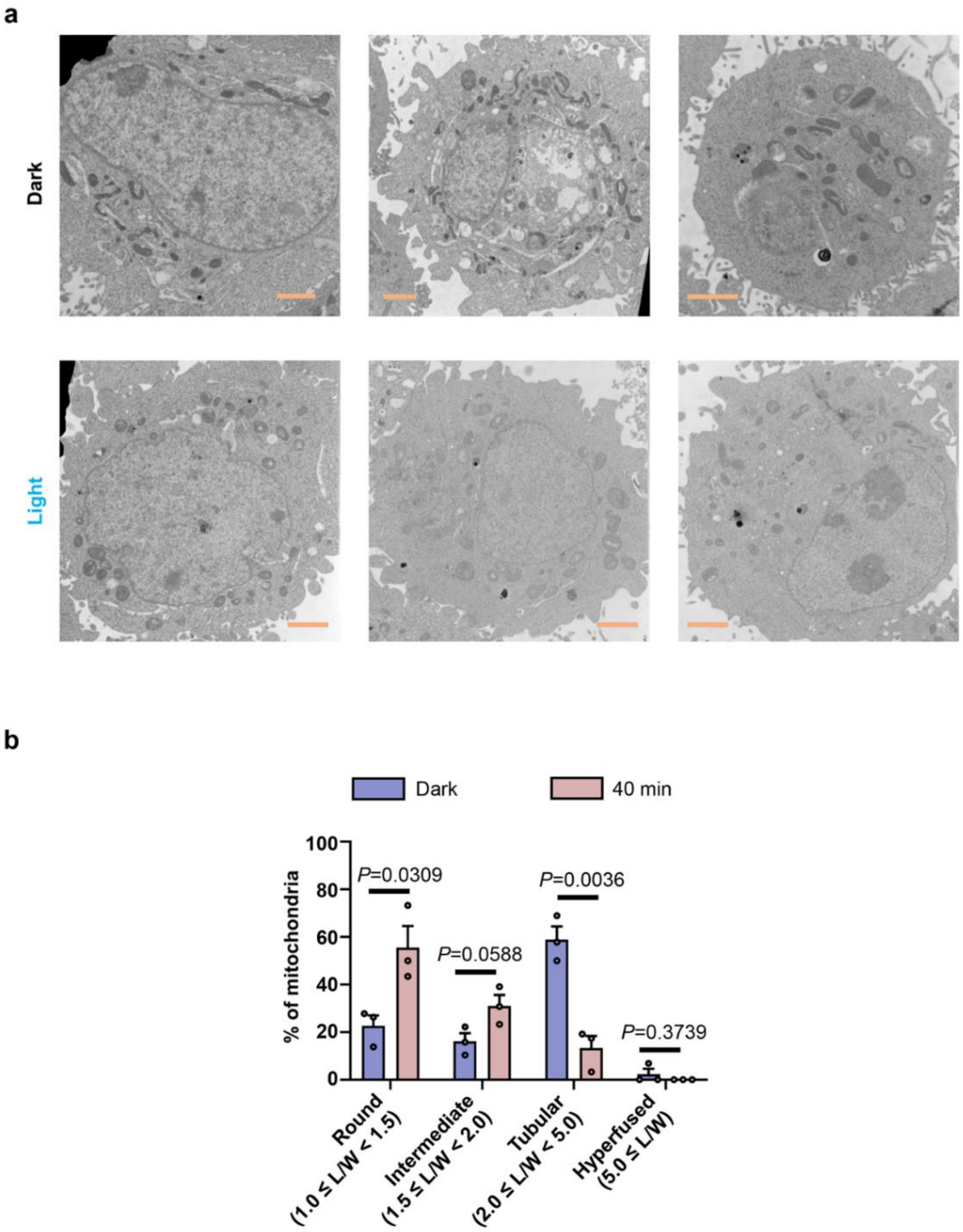

Supplementary Fig. 14 (**a**) The TEM images of HeLa cells expressing *LAMP–mCherry–CRY2* and *TOM20–CIB–GFP* with or without light exposure for 40 min. Scale bar: 2 μm. (**b**) Quantitative analysis of mitochondrial morphology of (a). Data are presented as $M \pm SEM$ from 3 independent experiments. The statistical differences between the experimental groups were analyzed by double-tailed Student's *t* test. When $P < 0.05$, it was considered to have statistical significance. Source data are provided as a Source Data file.

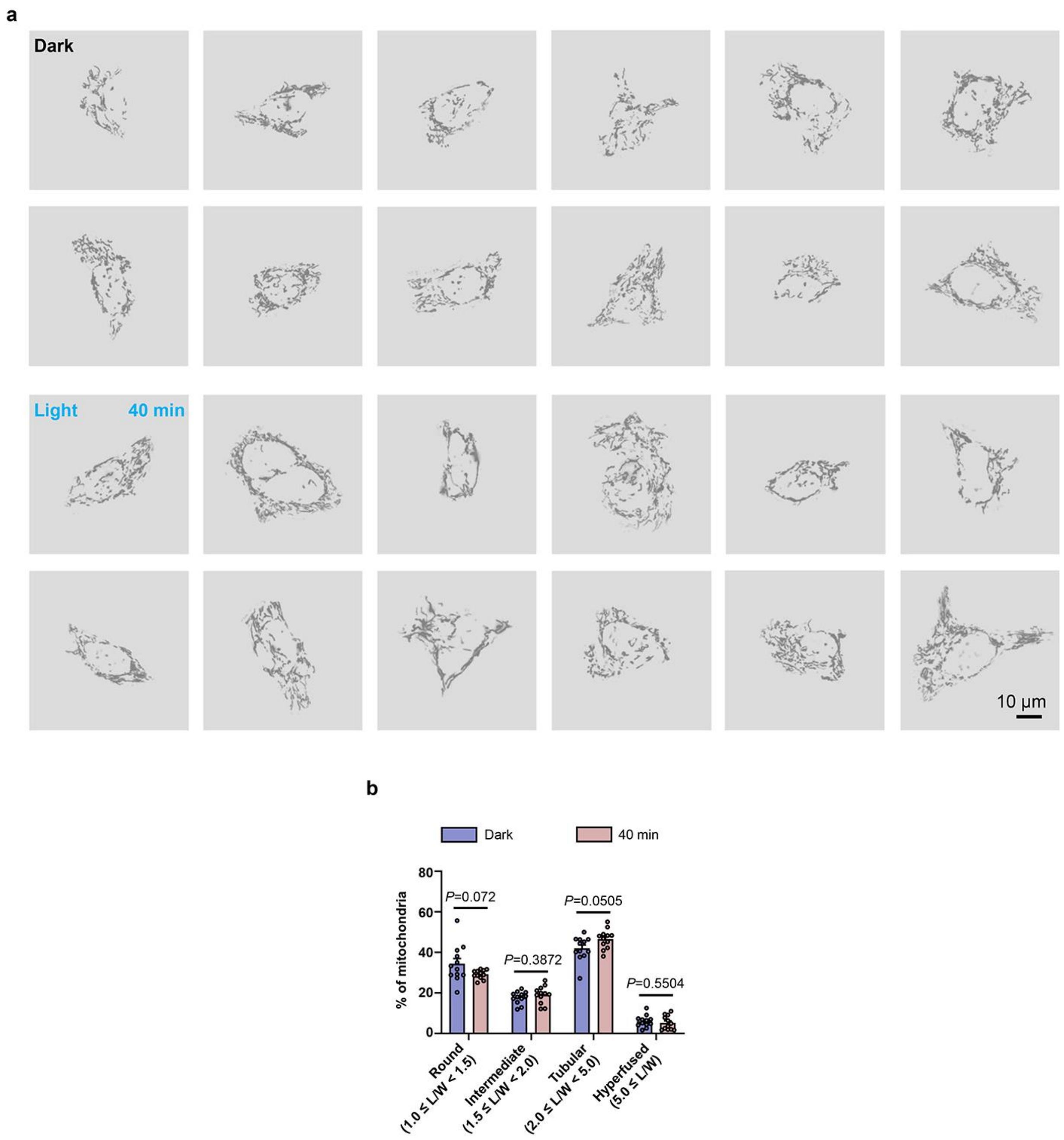

Supplementary Fig. 15 (**a**) The SIM images of mitochondria in living HeLa cells expressing *LAMP–mCherry–CRY2* and stained with MTG with or without blue light illumination. All images shared the same scale bar. (**b**) Quantitative analysis of mitochondrial morphology of (a). $n = 12$ cells examined over 3 independent experiments. Data are presented as $M \pm SEM$. The statistical differences between the experimental groups were analyzed by double-tailed Student's t test. When $P < 0.05$, it was considered to have statistical significance. Source data are provided as a Source Data file.

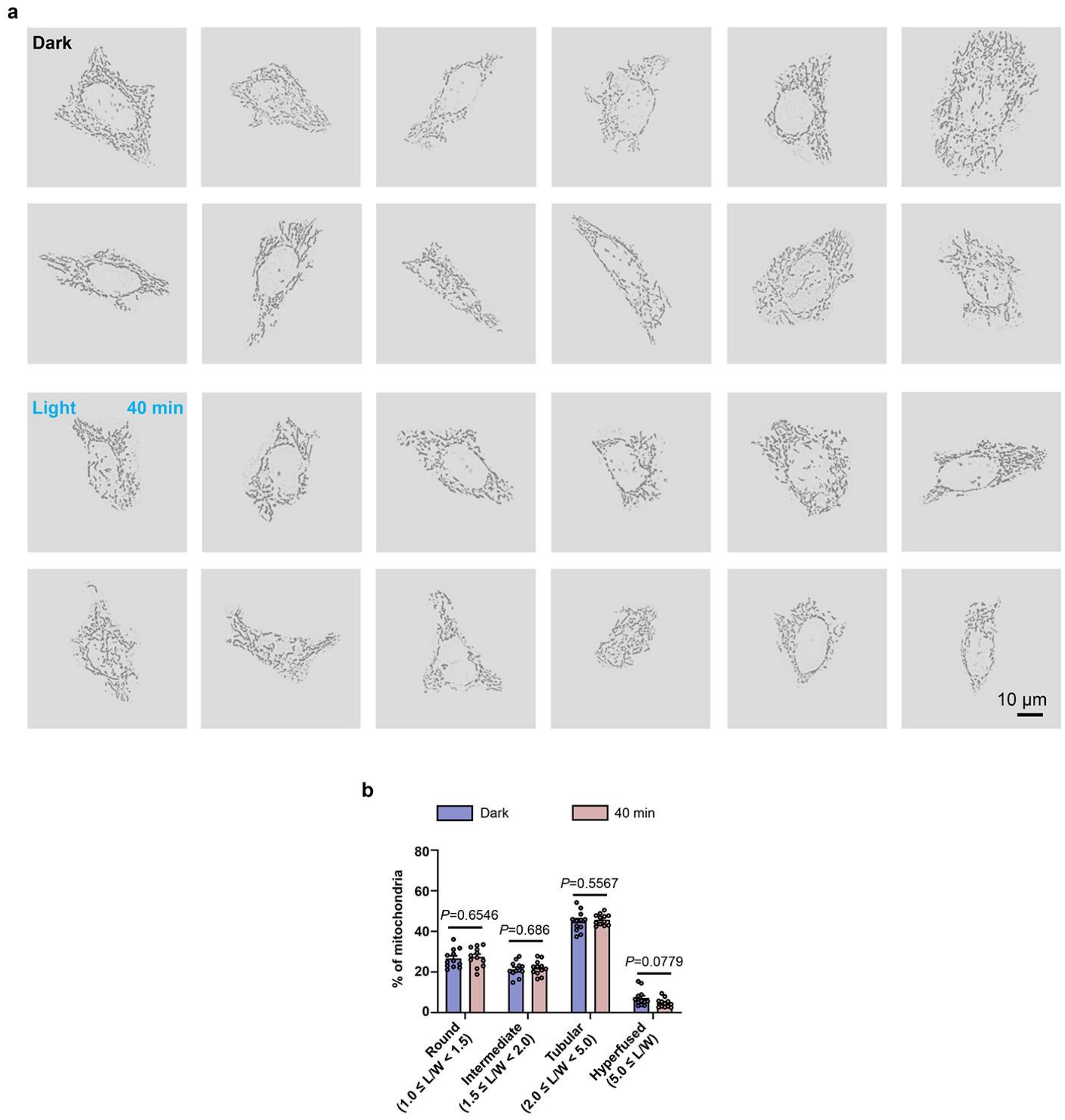

Supplementary Fig. 16 (**a**) The SIM images of mitochondria in living HeLa cells expressing *TOM20–CIB–GFP* and stained with MTR with or without blue light illumination. All images shared the same scale bar. (**b**) Quantitative analysis of mitochondrial morphology of (a). n = 12 cells examined over 3 independent experiments. Data are presented as $M \pm SEM$. The statistical differences between the experimental groups were analyzed by double-tailed Student's t test. When $P < 0.05$, it was considered to have statistical significance. Source data are provided as a Source Data file.

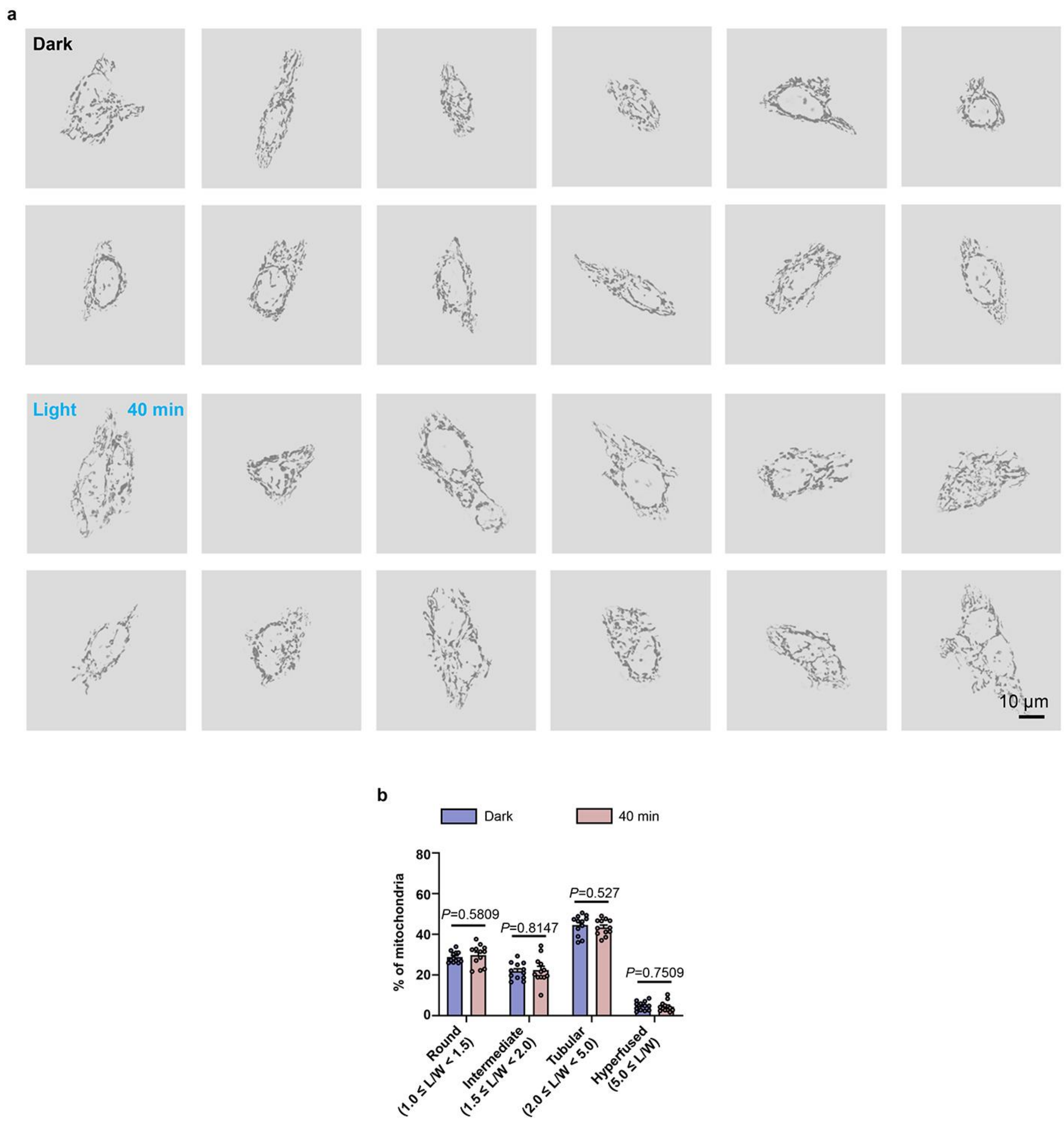

Supplementary Fig. 17 (**a**) The SIM images of mitochondria in living HeLa cells stained with MTG under blue-light illumination for different times. All images shared the same scale bar. (**b**) Quantitative analysis of mitochondrial morphology of (a). n = 12 cells examined over 3 independent experiments. Data are presented as $M \pm SEM$. The statistical differences between the experimental groups were analyzed by double-tailed Student's t test. When $P < 0.05$, it was considered to have statistical significance. Source data are provided as a Source Data file.

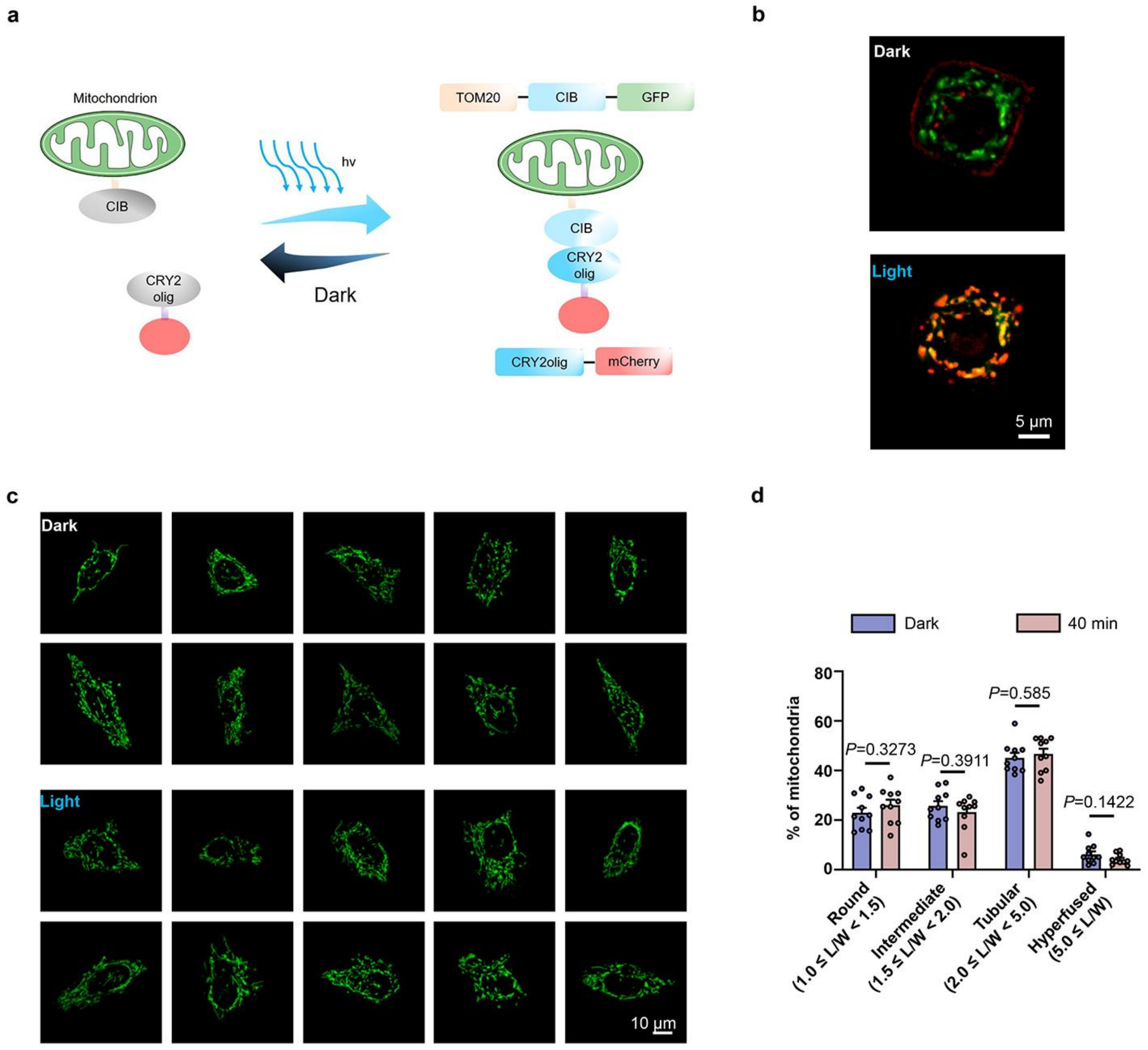

Supplementary Fig. 18 (**a**) Schematic representation of the contact of *CRY2olig-mCherry* and *TOM20-CIB-GFP* under blue light exposure. (**b**) Representative SIM images of mitochondria (green) and *CRY2olig-mCherry* (red) with or without blue light exposure. The two images shared the same scale bar. (**c**) The SIM images of mitochondria in living HeLa cells with or without blue light exposure for 40 min. All images shared the same scale bar. (**d**) Quantitative analysis of mitochondrial morphology in (c). n = 10 cells examined over 3 independent experiments. Data are presented as $M \pm SEM$. The statistical differences between the experimental groups were analyzed by double-tailed Student's t test. When $P <$ 0.05, it was considered to have statistical significance. Source data are provided as a Source Data file.

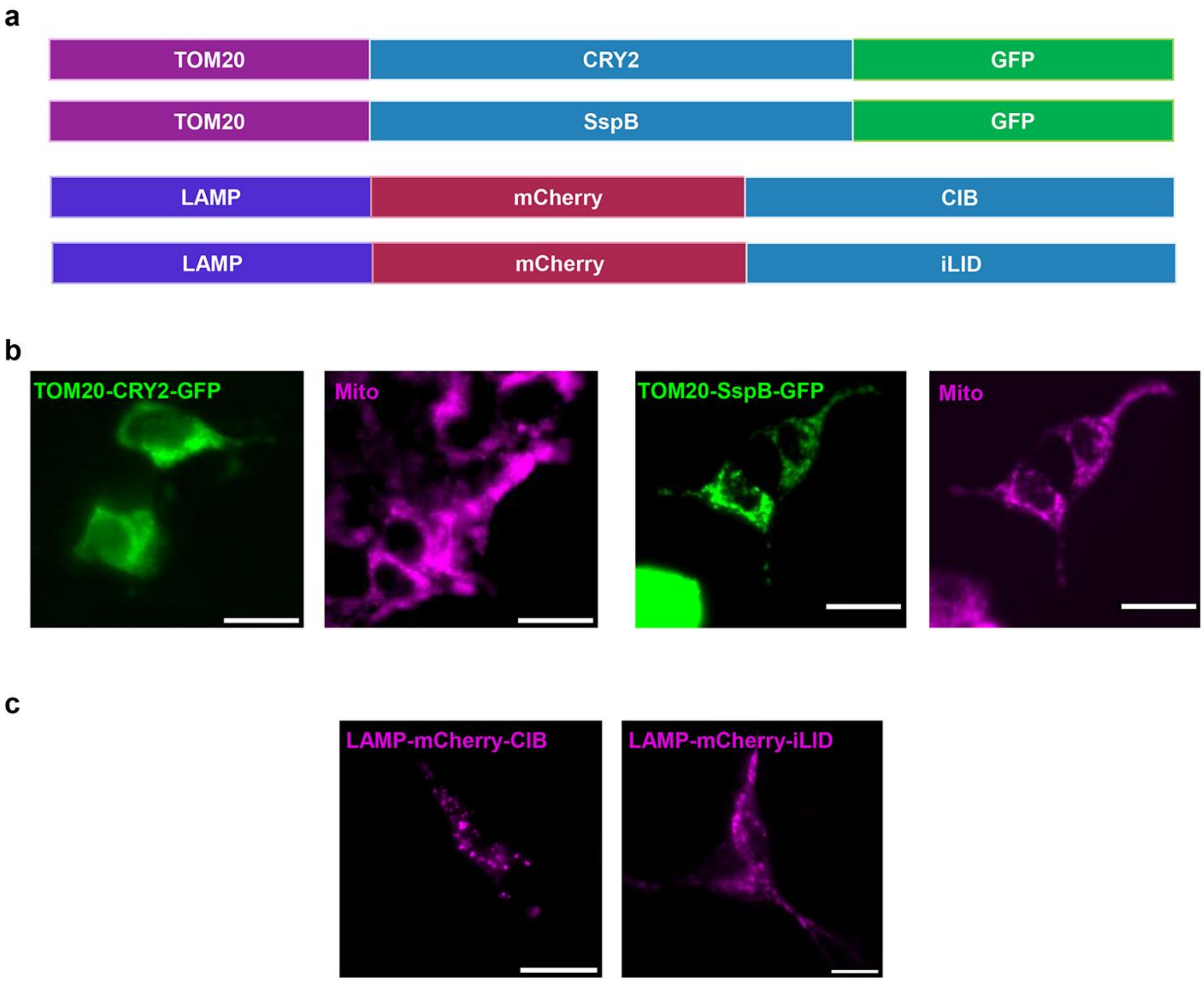

Supplementary Fig. 19 Construction of plasmids for studying light-induced mitochondrial fission via MLCs in different photoactivable systems. (**a**) Schematic depiction of the plasmids prepared for studying mitochondrial fission using MLCs using CRY2-CRY2, reversed CRY2-CIB and SspB-iLID based systems. (**b**) Representative epifluorescence images of mitochondria-targeted constructs fused with GFP shown in green (for *TOM20-CRY2-GFP* and *TOM20-SspB-GFP*) counter stained with MTR (magenta) for confirmation. (**c**) Representative epifluorescence images of lysosome-targeted constructs fused with mCherry in magenta (for *LAMP-mCherry-CIB* and *LAMP-mCherry-iLID*) to highlight vesicular morphology of the lysosomes for confirmation. Scale bar: 20 μm for all images.

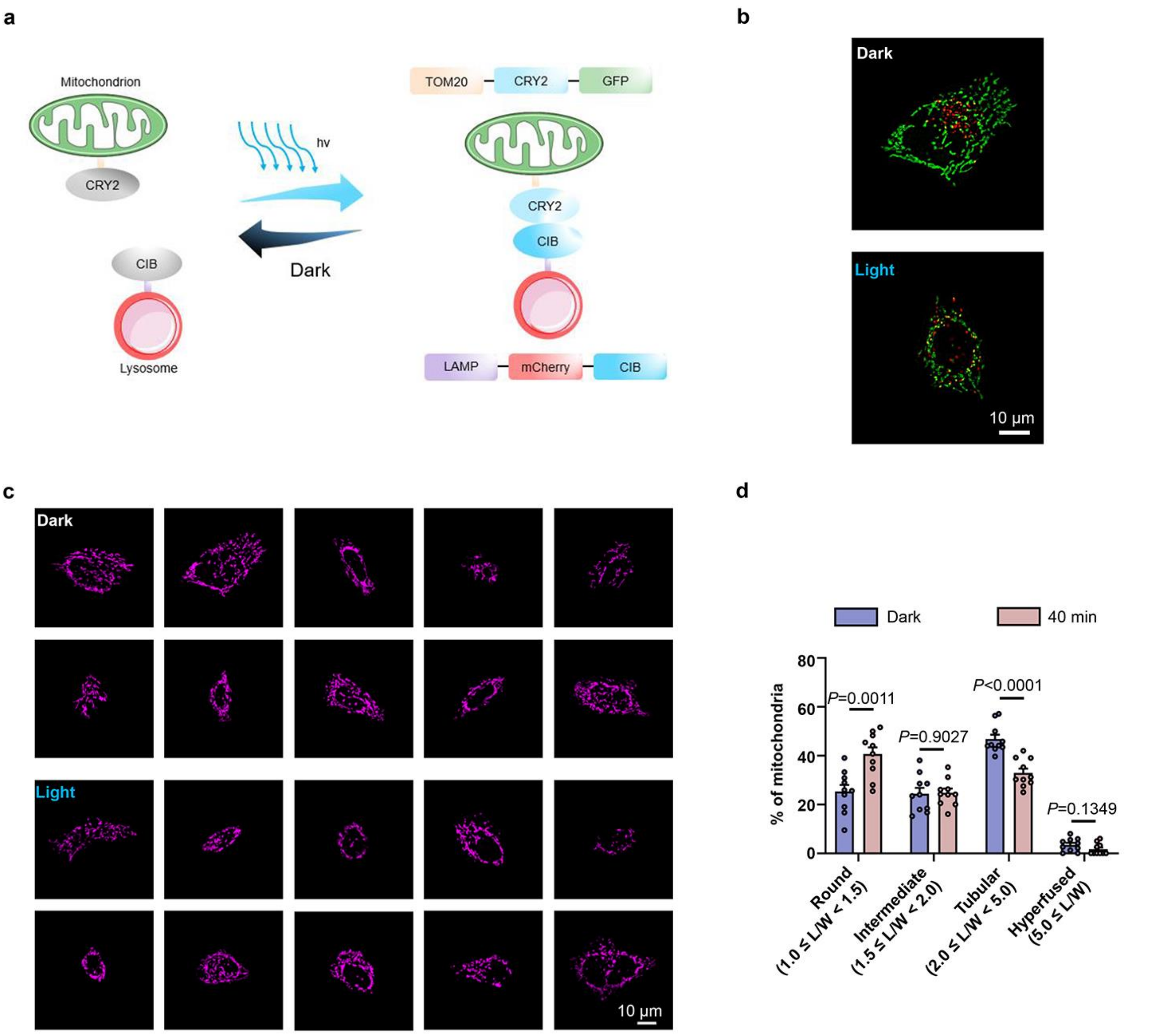

Supplementary Fig. 20 (**a**) Schematic representation of the contact of *LAMP–mCherry–CIB* and *TOM20–CRY2–GFP* under blue light exposure. (**b**) Representative SIM images of mitochondria (green) and lysosomes (red) with or without blue light exposure. Two images shared the same scale bar. (**c**) The SIM images of mitochondria in living HeLa cells expressing *LAMP–mCherry–CIB* and *TOM20–CRY2–GFP* and staining MTDR with or without blue light exposure for 40 min. All images shared the same scale bar. (**d**) Quantitative analysis of mitochondrial morphology in (c). n = 10 cells examined over 3 independent experiments. Data are presented as $M \pm SEM$. The statistical differences between the experimental groups were analyzed by double-tailed Student's t test. When $P < 0.05$, it was considered to have statistical significance. Source data are provided as a Source Data file.

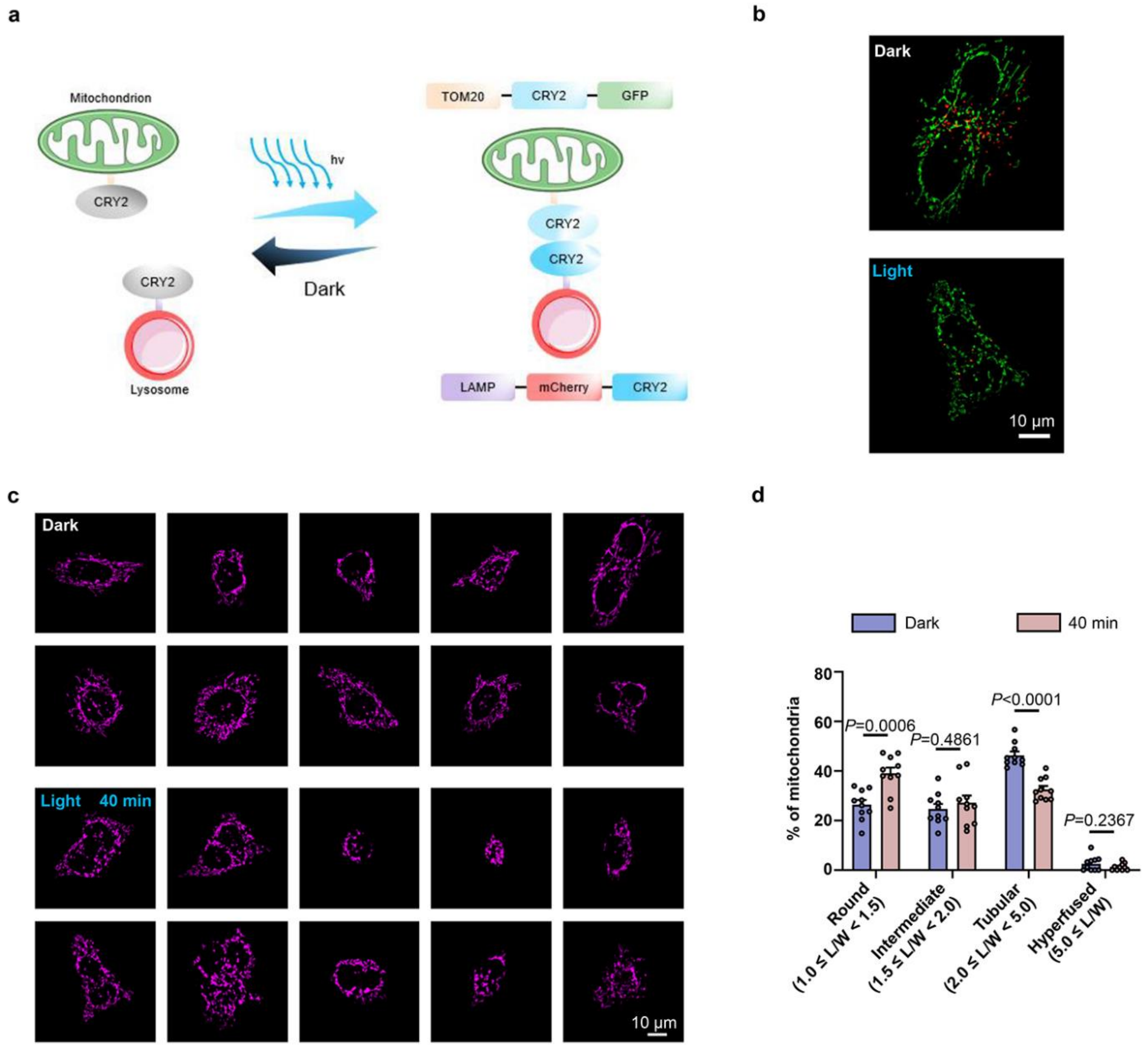

Supplementary Fig. 21 (**a**) Schematic representation of the contact of *LAMP–mCherry–CRY2* and *TOM20–CRY2–GFP* under blue light exposure. (**b**) Representative SIM images of mitochondria (green) and lysosomes (red) with or without blue light exposure. Two images shared the same scale bar. (**c**) The SIM images of mitochondria in living HeLa cells expressing *LAMP–mCherry–CRY2* and *TOM20–CRY2–GFP* and staining MTDR with or without blue light exposure for 40 min. All images shared the same scale bar. (**d**) Quantitative analysis of mitochondrial morphology in (c). $n = 10$ cells examined over 3 independent experiments. Data are presented as $M \pm SEM$. The statistical differences between the experimental groups were analyzed by double-tailed Student's t test. When $P < 0.05$, it was considered to have statistical significance. Source data are provided as a Source Data file.

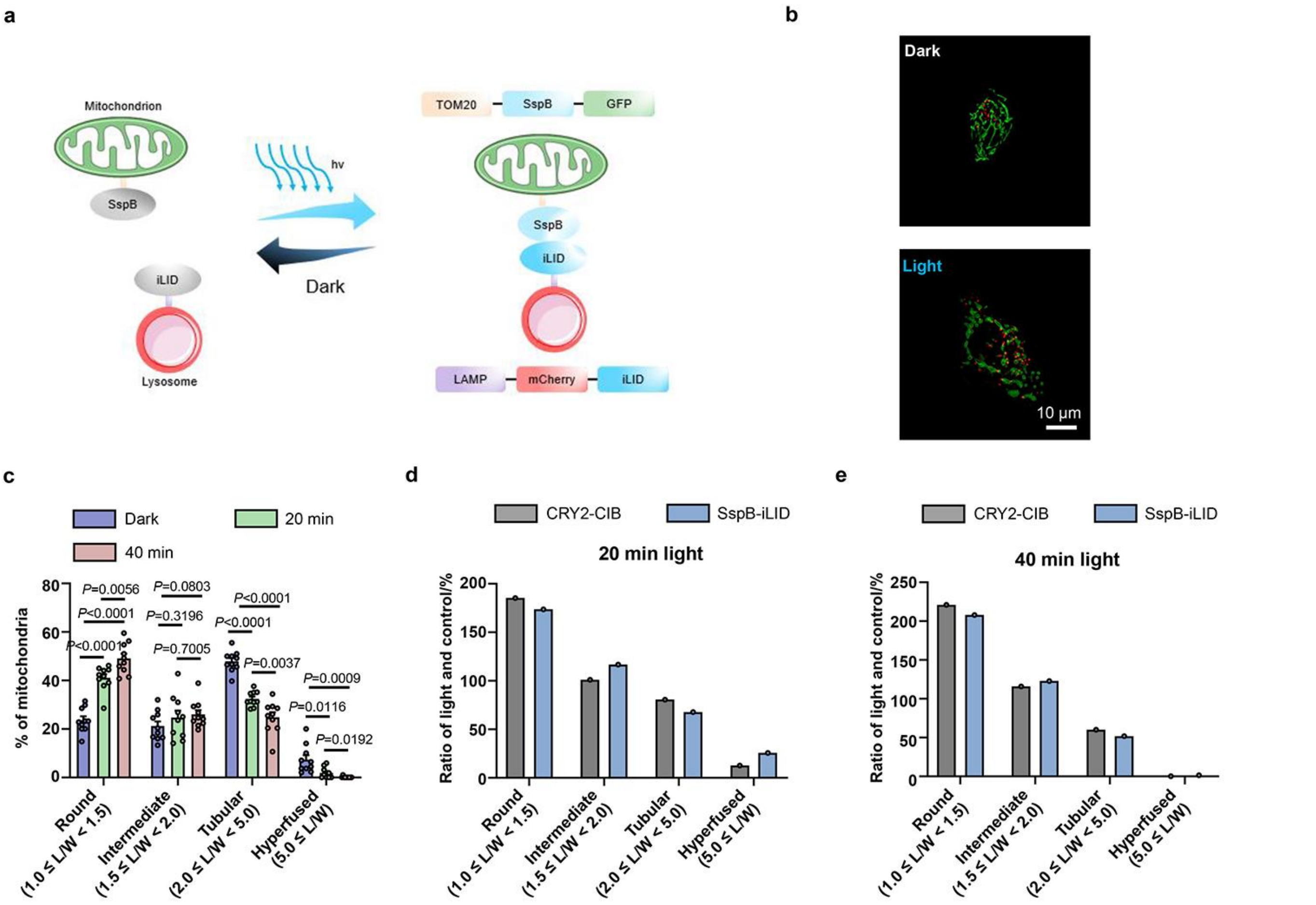

Supplementary Fig. 22 (**a**) Schematic representation of the contact of *LAMP–mCherry–iLID* and *TOM20–SspB–GFP* under blue light exposure. (**b**) Representative SIM images of mitochondria (green) and lysosomes (red) with or without blue light exposure. The two images shared the same scale bar. (**c**) Quantitative analysis of mitochondrial morphology for HeLa cells expressing *LAMP–mCherry–iLID* and *TOM20–SspB–GFP* under different durations of blue light exposure. n = 10 cells examined over 3 independent experiments. Data are presented as $M \pm SEM$. The statistical differences between the experimental groups were analyzed by double-tailed Student's t test. When $P < 0.05$, it was considered to have statistical significance. The kinetic for mitochondrial fission between CRY2–CIB (*LAMP–mCherry–CRY2* and *TOM20–CIB–GFP*) and SspB–iLID (*TOM20–SspB–GFP* and *LAMP–mCherry–iLID*) under blue light for 20 min **(d)** and 40 min (**e**). Source data are provided as a Source Data file.

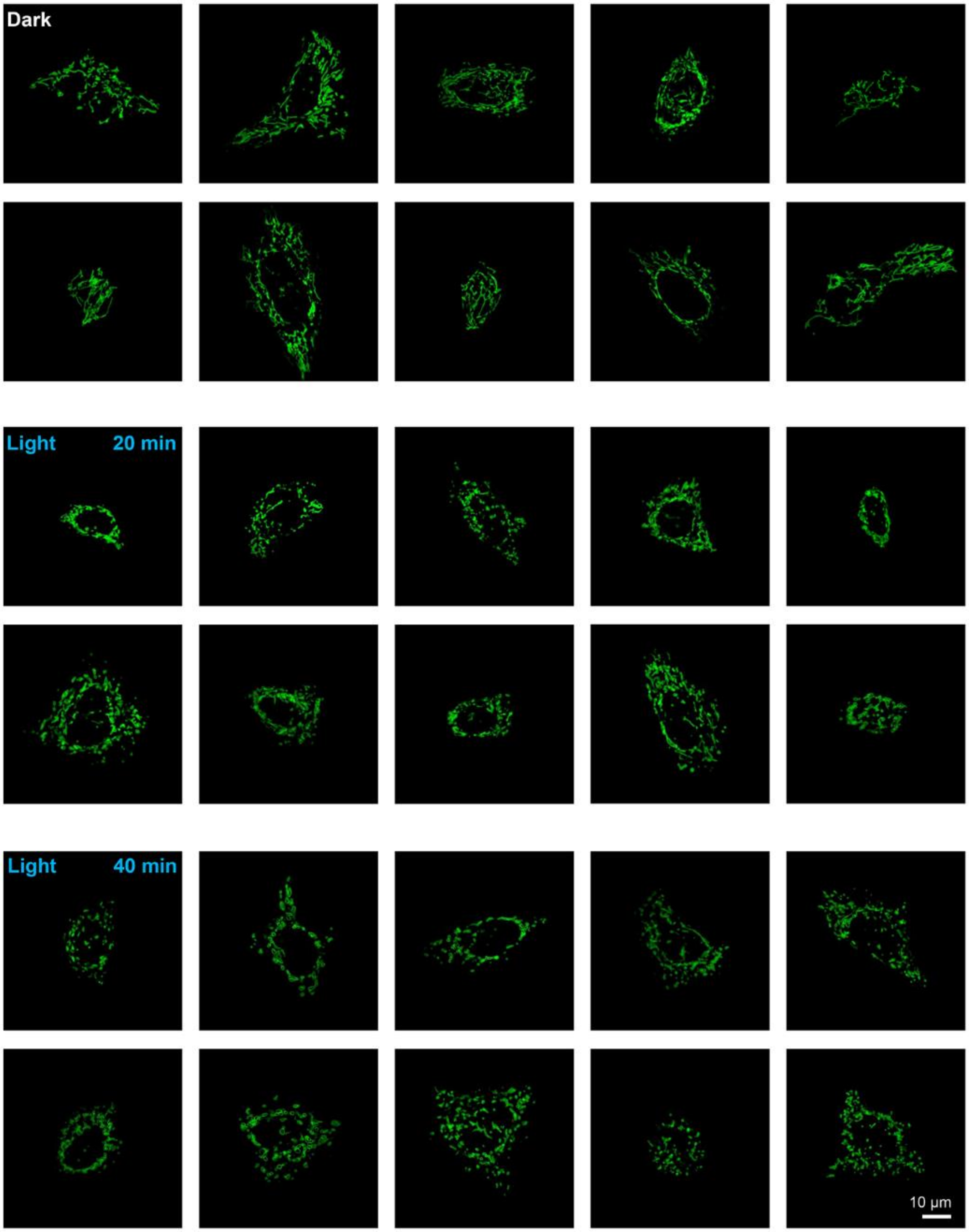

Supplementary Fig. 23 The data set for Supplementary Fig. 22c. The SIM images of mitochondrial morphology for HeLa cells expressing *LAMP–mCherry–iLID* and *TOM20–SspB–GFP* under different durations of blue light exposure. All images shared the same scale bar.

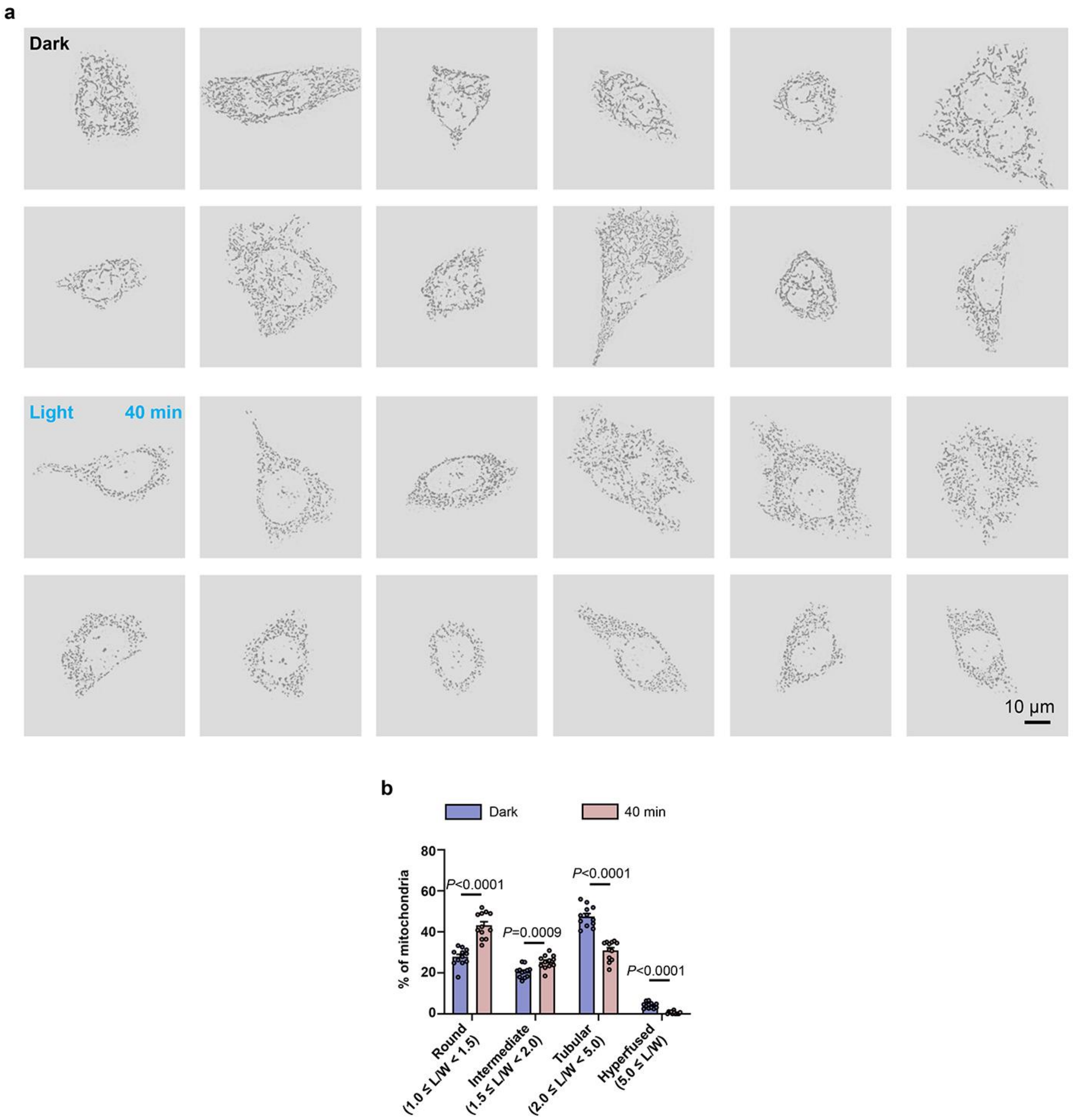

Supplementary Fig. 24 The size reduction of mitochondria in living PC12 cells induced by optogenetic MLCs system. (**a**) The SIM images of mitochondria in PC12 cells expressing *LAMP–CRY2* and *TOM20–CIB–GFP*, and stained with MTR under blue-light illumination for 0 or 40 min. All images shared the same scale bar. (**b**) Quantitative analysis of mitochondrial morphology of (a). n = 12 cells examined over 3 independent experiments. Data are presented as $M \pm SEM$. The statistical differences between the experimental groups were analyzed by double-tailed Student's t test. When $P < 0.05$, it was considered to have statistical significance. Source data are provided as a Source Data file.

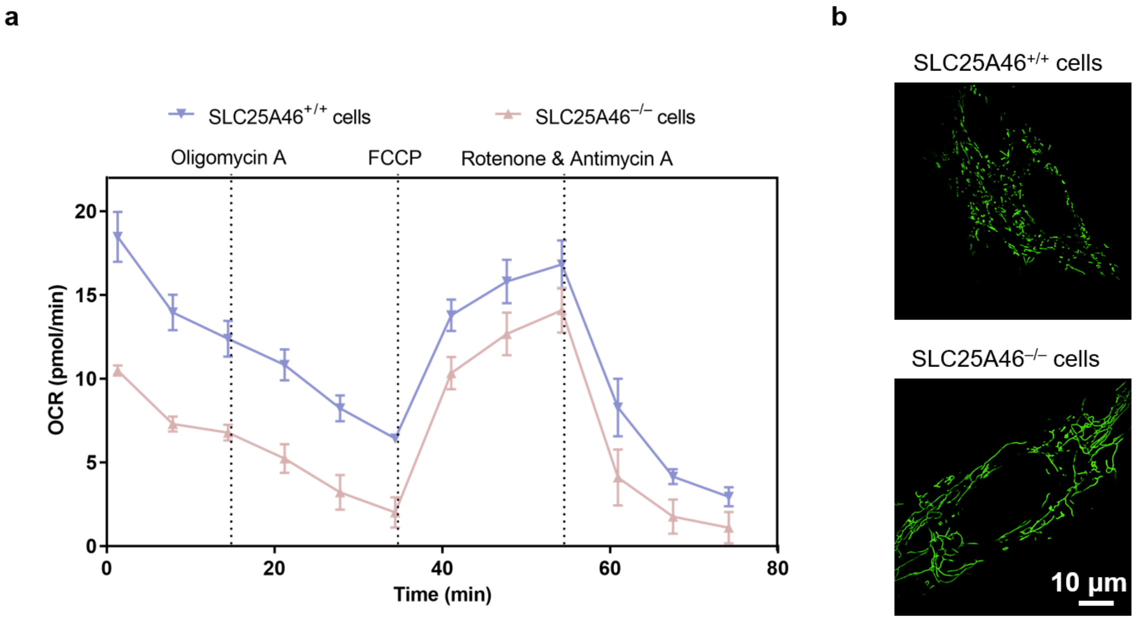

Supplementary Fig. 25 (**a**) OCR curves and (**b**) mitochondrial images of wild-type and SLC25A46$^{-/-}$ HDFn cells. Two images shared the same scale bar. The OCR curves from n = 4 biologically independent experiments. Data are presented as $M \pm SEM$. Source data are provided as a Source Data file.

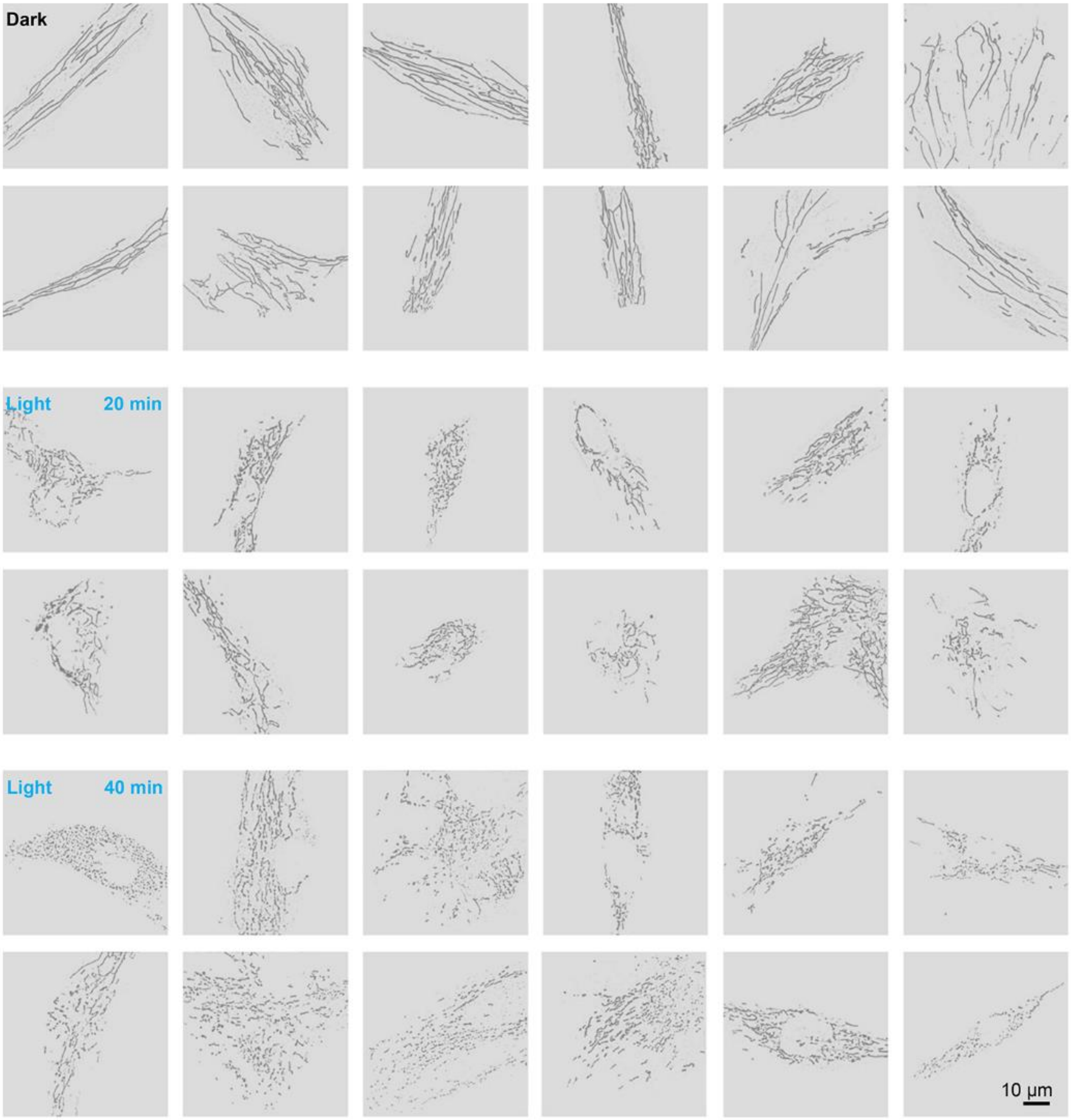

Supplementary Fig. 26 The data set for Fig. 3b. The SIM images of mitochondria with different blue-light stimulation time (0, 20, and 40 min) in living SLC25A46$^{-/-}$ HDFn cells expressing *LAMP–CRY2* and *TOM20–CIB–GFP*, and stained with MTR. All images shared the same scale bar.

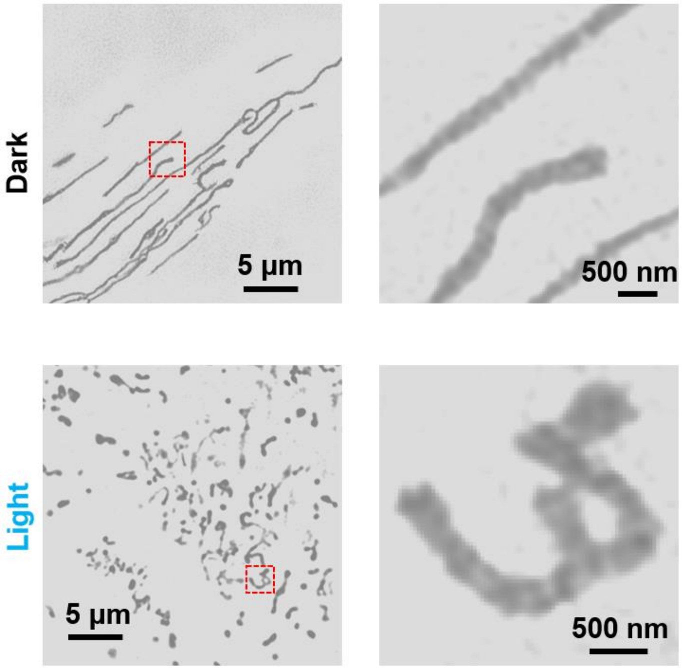

Supplementary Fig. 27 The SIM images of mitochondria cristae in SLC25A46$^{-/-}$ cells expressing *LAMP–CRY2* and *TOM20–CIB–GFP*, and stained with MTR in dark or with 20 min blue light exposure.

	Optogenetic method	Chemical method	Biological method
**Reversibility**	√	×	×
**Spatiotemporal controllability**	√	×	×
**Safety**	√	×	√
**Integrity of cellular proteins**	√	√	×

Supplementary Fig. 28 Comparison of methods for mitochondrial fission in term of reversibility, spatiotemporal controllability and safety.

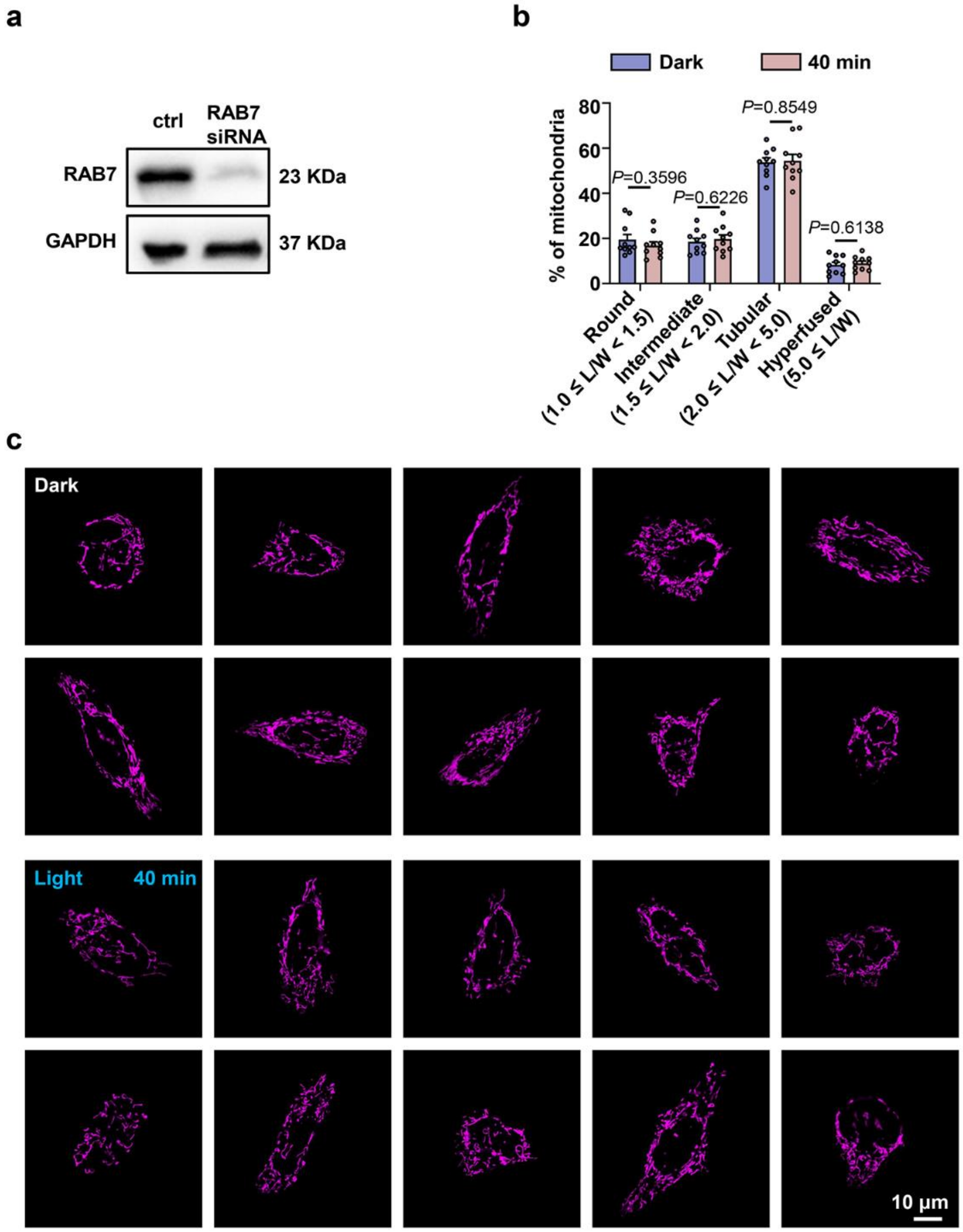

Supplementary Fig. 29 (**a**) The protein bands of western blotting assay with siRNA knock-down (KD) of RAB7. (**b**) Quantitative analysis of mitochondrial morphology for RAB7 KD HeLa cells expressing *LAMP–mCherry–CRY2* and *TOM20–CIB–GFP* without or with blue light exposure for 40 min. n = 10 cells examined over 3 independent experiments. Data are presented as $M \pm SEM$. The statistical differences between the experimental groups were analyzed by double-tailed Student's t test. When $P < 0.05$, it was considered to have statistical significance. (**c**) The SIM images of mitochondria analyzed in (**b**). All images shared the same scale bar. Source data are provided as a Source Data file.

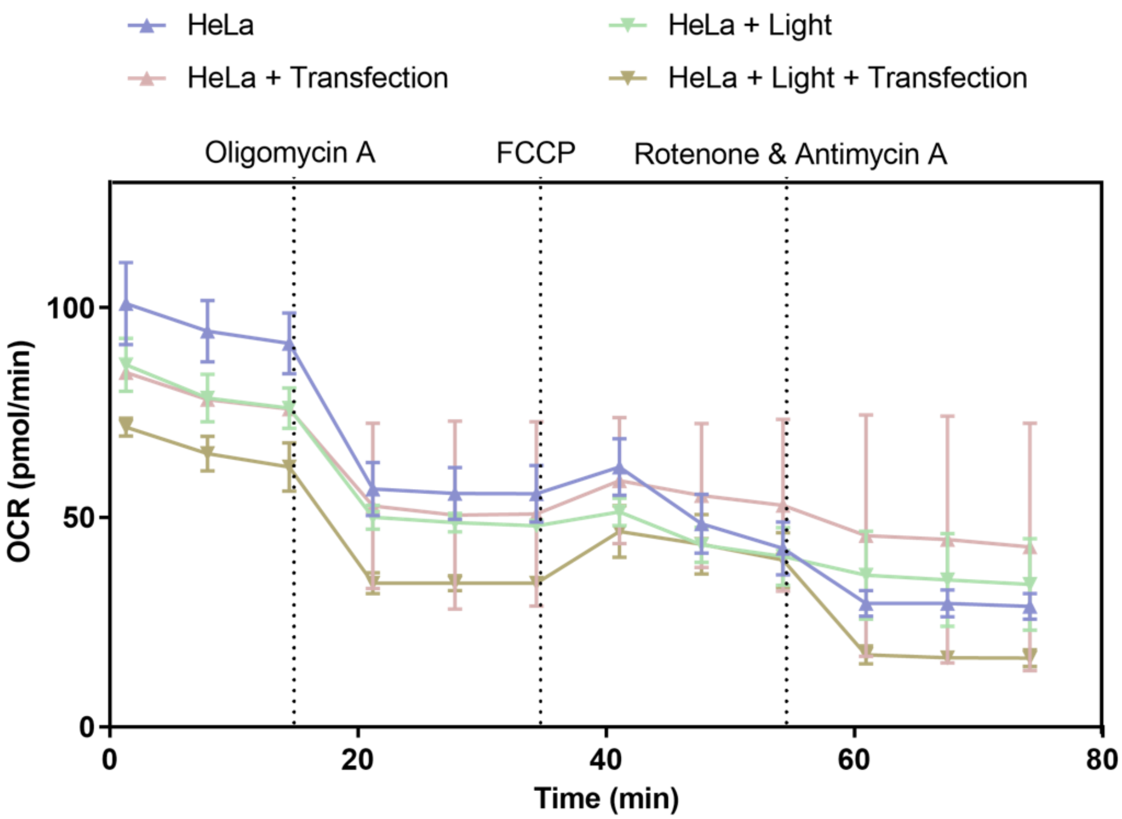

Supplementary Fig. 30 Effects on aerobic respiration in HeLa cells with optogenetic MLCs system and 20 min blue-light illumination. OCR before the addition of oligomycin A indicates the basal respiration; OCR after the injection of FCCP denotes the maximal mitochondrial respiration capacity; and OCR after the injection of rotenone and antimycin A shows non-mitochondrial respiration. Due to disruption of mitochondrial dynamics, the OCR level in basal respiration decreased after the optogenetic treatment. The OCR curves from n = 3 biologically independent experiments. Data are presented as $M \pm SEM$. Source data are provided as a Source Data file.

a

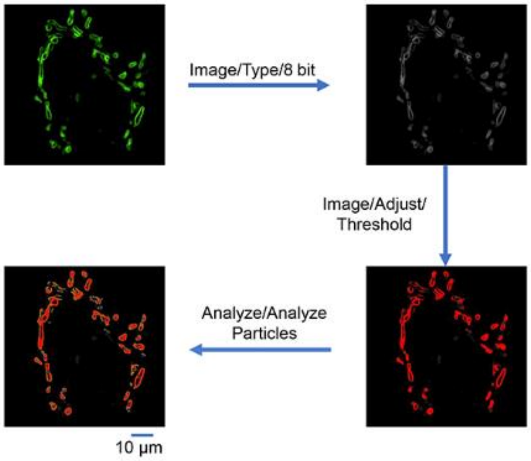

	L/W		L/W		L/W
**1**	1	**21**	1.681	**41**	2.824
**2**	1.041	**22**	1.691	**42**	3.017
**3**	1.113	**23**	1.694	**43**	3.236
**4**	1.158	**24**	1.695	**44**	3.475
**5**	1.176	**25**	1.811	**45**	3.553
**6**	1.179	**26**	1.894	**46**	3.58
**7**	1.209	**27**	1.956	**47**	3.614
**8**	1.25	**28**	2.013	**48**	3.779
**9**	1.288	**29**	2.03	**49**	4.825
**10**	1.362	**30**	2.05	**50**	4.935
**11**	1.416	**31**	2.107	**51**	5.268
**12**	1.489	**32**	2.224	**52**	5.337
**13**	1.499	**33**	2.274	**53**	5.646
**14**	1.518	**34**	2.338	**54**	9.22
**15**	1.542	**35**	2.367		
**16**	1.553	**36**	2.386		
**17**	1.572	**37**	2.483		
**18**	1.635	**38**	2.485		
**19**	1.638	**39**	2.776		
**20**	1.657	**40**	2.81		

b

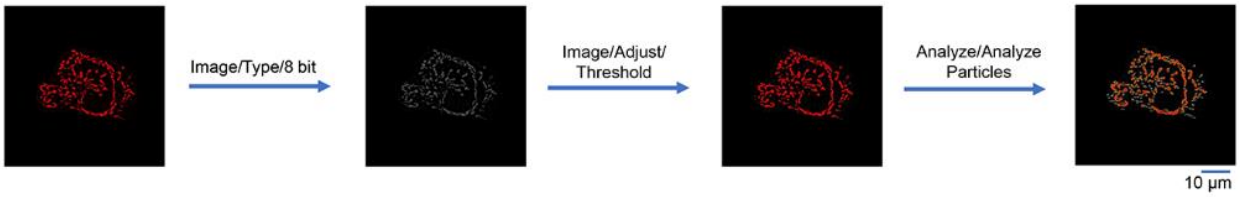

	L/W		L/W		L/W		L/W		L/W		L/W
**1**	1	**21**	1.372	**41**	1.613	**61**	2.031	**81**	2.517	**101**	3.495
**2**	1	**22**	1.372	**42**	1.615	**62**	2.043	**82**	2.581	**102**	3.545
**3**	1	**23**	1.408	**43**	1.635	**63**	2.054	**83**	2.636	**103**	3.582
**4**	1	**24**	1.437	**44**	1.647	**64**	2.08	**84**	2.722	**104**	3.598
**5**	1.017	**25**	1.455	**45**	1.649	**65**	2.094	**85**	2.726	**105**	3.688
**6**	1.02	**26**	1.464	**46**	1.675	**66**	2.101	**86**	2.793	**106**	3.787
**7**	1.087	**27**	1.472	**47**	1.681	**67**	2.195	**87**	2.833	**107**	3.979
**8**	1.092	**28**	1.476	**48**	1.691	**68**	2.213	**88**	2.844	**108**	4.173
**9**	1.113	**29**	1.498	**49**	1.711	**69**	2.221	**89**	2.87	**109**	4.212
**10**	1.154	**30**	1.507	**50**	1.766	**70**	2.258	**90**	2.947	**110**	4.348
**11**	1.186	**31**	1.515	**51**	1.78	**71**	2.277	**91**	2.962	**111**	4.479
**12**	1.226	**32**	1.526	**52**	1.795	**72**	2.278	**92**	2.985	**112**	4.588
**13**	1.226	**33**	1.529	**53**	1.817	**73**	2.315	**93**	3.058	**113**	4.643
**14**	1.229	**34**	1.543	**54**	1.85	**74**	2.349	**94**	3.158	**114**	4.999
**15**	1.255	**35**	1.552	**55**	1.869	**75**	2.359	**95**	3.16	**115**	5.017
**16**	1.257	**36**	1.553	**56**	1.907	**76**	2.419	**96**	3.186	**116**	5.115
**17**	1.297	**37**	1.553	**57**	1.947	**77**	2.457	**97**	3.199	**117**	5.304
**18**	1.318	**38**	1.562	**58**	2	**78**	2.461	**98**	3.321	**118**	5.507
**19**	1.319	**39**	1.576	**59**	2	**79**	2.49	**99**	3.374	**119**	7.992
**20**	1.327	**40**	1.609	**60**	2	**80**	2.512	**100**	3.402		

Supplementary Fig. 31 Comparison of (**a**) GFP and (**b**) MTR for the quantitative analysis of mitochondrial morphology by ImageJ. Since the GFP fluorescence is on the mitochondrial membrane, the ImageJ software could interpret the width as the width of the mitochondrial membrane and the length as the perimeter of the mitochondrial membrane. MTR stains the whole mitochondrion and its fluorescence represents the full morphology of mitochondria. Therefore, using small MitoTrackers MTR, MTG, and MTDR for quantitative analysis of mitochondrial morphology is more reliable.

Supplementary Table 1 The quantification data of mitochondrial morphology for HeLa cells expressing *LAMP–mCherry–CRY2* and *TOM20–CIB–GFP* under blue light exposure at different time points (min)

**Round** (1.0 ≤ L/W < 1.5)/%												
**0**	20.29	22.22	7.41	21.74	19.045	10.00	28.30	30.00	17.24	24.56	10.00	26.32
**20**	29.41	22.62	31.58	25.93	45.00	53.57	46.67	40.91	32.14	38.33	40	34.88
**40**	50.00	41.86	51.61	48.10	40.00	27.78	37.84	42.11	37.50	46.73	49.06	51.92
**Intermediate** (1.5 ≤ L/W < 2.0)/%												
**0**	15.94	22.22	22.22	26.09	23.81	30.00	28.30	23.33	31.03	28.07	20.00	21.05
**20**	35.29	27.38	10.53	33.33	21.67	14.29	28.33	13.64	32.14	23.33	18.18	32.56
**40**	26.67	30.23	22.58	24.05	28.33	38.89	37.84	21.05	25.00	28.97	26.42	28.85
**Tubular** (2.0 ≤ L/W < 5.0)/%												
**0**	57.97	50.00	59.26	47.83	53.97	45.00	37.74	40.00	44.83	42.11	45.00	36.84
**20**	35.29	46.43	57.89	37.04	30.00	32.14	25.00	45.45	35.71	35.00	41.82	27.91
**40**	23.33	27.91	25.81	27.85	31.67	33.33	24.32	36.84	37.50	24.30	24.53	19.23
**Hyperfused** (5.0 ≤ L/W)/%												
**0**	5.8	5.56	11.11	4.35	3.17	15.00	5.66	6.67	6.90	5.26	25	15.79
**20**	0	3.57	0	3.70	3.33	0	0	0	0	3.33	0	4.65
**40**	0	0	0	0	0	0	0	0	0	0	0	0

Supplementary Table 2 The quantification data of mitochondrial morphology for PC12 cells expressing *LAMP–mCherry–CRY2* and *TOM20–CIB–GFP* under blue light exposure at different time points (min)

**Round** (1.0 ≤ L/W < 1.5)/%												
**0**	25.57	24.80	24.91	26.28	27.90	32.54	30.10	29.36	29.93	17.91	31.27	33.33
**40**	49.79	42.47	48.94	48.41	40.83	51.92	40.88	49.06	36.42	39.85	36.40	33.51
**Intermediate** (1.5 ≤ L/W < 2.0)/%												
**0**	21.02	16.00	21.45	17.95	25.46	20.63	25.26	20.18	18.25	21.64	17.57	21.26
**40**	23.85	22.58	26.06	18.47	28.44	26.54	23.36	23.60	26.94	25.29	28.31	30.89
**Tubular** (2.0 ≤ L/W < 5.0)/%												
**0**	48.30	54.40	50.91	51.92	44.20	40.48	41.52	45.87	45.26	55.97	48.06	43.00
**40**	26.36	34.95	25.00	31.21	30.73	21.54	35.04	27.34	35.56	34.10	34.56	34.55
**Hyperfused** (5.0 ≤ L/W)/%												
**0**	5.11	4.80	2.73	3.85	2.44	6.35	3.11	4.59	6.57	4.48	3.10	2.42
**40**	0	0	0	1.91	0	0	0.73	0	1.078	0.77	0.74	1.05

Supplementary Table 3 The quantification data of mitochondrial morphology for SLC25A46$^{-/-}$ HDFn cells expressing *LAMP–mCherry–CRY2* and *TOM20–CIB–GFP* under blue light exposure at different time points (min)

**Round** (1.0 ≤ L/W < 1.5)/%												
**0**	20.83	11.67	20.00	13.97	10.00	21.43	10.53	6.90	15.94	26.44	11.86	2.70
**20**	24.70	21.43	24.51	29.27	24.16	19.82	22.22	18.97	24.44	22.66	23.16	22.58
**40**	29.63	28.97	32.78	32.77	28.74	30.65	34.18	32.16	30.05	35.70	40.46	36.36
**Intermediate** (1.5 ≤ L/W < 2.0)/%												
**0**	8.33	10.00	6.96	5.88	15.00	5.49	9.21	13.79	18.84	6.90	11.86	5.41
**20**	15.85	19.39	24.51	20.33	16.11	26.13	20.00	12.07	22.22	24.22	23.68	21.51
**40**	18.06	23.93	25.52	21.01	20.96	20.16	17.72	21.60	19.69	20.51	25.57	21.02
**Tubular** (2.0 ≤ L/W < 5.0)/%												
**0**	30.56	43.33	33.91	50.00	10.00	45.60	48.68	48.28	36.23	40.23	27.12	37.84
**20**	50.61	46.94	45.10	40.65	46.98	45.05	46.67	46.55	48.89	40.62	47.89	43.01
**40**	42.59	39.55	37.76	42.02	40.72	39.52	42.41	43.19	38.86	34.43	33.10	38.64
**Hyperfused** (5.0 ≤ L/W)/%												
**0**	40.28	35.00	39.13	30.15	65.00	27.47	31.58	31.03	28.99	26.44	49.15	54.05
**20**	8.84	12.24	5.88	9.76	12.75	9.01	11.11	22.41	4.44	12.50	5.26	12.90
**40**	9.72	7.56	3.94	4.20	9.58	9.68	5.70	3.05	11.40	9.37	0.88	3.98
