# Supplementary material for: Light-activated mitochondrial fission through optogenetic control of mitochondria-lysosome contacts
Source: Nat Commun. 2022 Jul 25;13:4303. doi: 10.1038/s41467-022-31970-5 (PMC9314359; doi:10.1038/s41467-022-31970-5)
Supplement: Supplementary file 3 — Description of additional Supplementary Information [file 41467_2022_31970_MOESM3_ESM.docx]

**Inventory of Supporting Information**

**Supplementary Information file**

Supplementary Figures 1-31

Supplementary Tables 1-3

**Supplementary Movie**

Supplementary Movie 1

Description: The real-time tracking of the blue light-induced association between CIB and CRY2PHR fusion proteins with 2 second/frame in BHK21 cells expressing CIB-GFP-CAAX and CRY2PHR-mCherry-Raf1.

Supplementary Movie 2

Description: The real-time super-resolution tracking of the contact between lysosomes and mitochondria at the site of mitochondrial division (white arrows) during mitochondrial fission in living HeLa cells expressing LAMP–mCherry–CRY2 (i.e., for lysosomes) and TOM20–CIB–GFP (i.e., for mitochondria) with time intervals of 20 s under blue light illumination.

Supplementary Movie 3

Description: The real-time tracking of the interplay between lysososomes and mitochondria in a HeLa cell expressing LAMP–mCherry–CRY2 and TOM20–CIB–GFP with time intervals of 20 s under blue light exposure.
