# Supplementary material for: Light-activated mitochondrial fission through optogenetic control of mitochondria-lysosome contacts
Source: Nat Commun. 2022 Jul 25;13:4303. doi: 10.1038/s41467-022-31970-5 (PMC9314359; doi:10.1038/s41467-022-31970-5)
Supplement: Supplementary file 2 — Editorial Assessment Report [file 41467_2022_31970_MOESM2_ESM.pdf]

nature portfolio

GUIDED OPEN ACCESS
nature methods | nature communications | communications biology

Editorial Assessment Report

Dear Professor Diao,

Thank you again for choosing to submit your manuscript using the Nature Portfolio Guided Open Access pilot. As part of this process, our Nature Portfolio editorial team has considered your paper for three of our journals with strong interest in publishing in your field: *Nature Methods*, *Nature Communications*, and *Communications Biology*.

Your manuscript entitled "Light-activated mitochondrial fission through optogenetic control of mitochondria-lysosome contacts" has now been reviewed by three experts in optogenetics, mitochondrial dynamics and metabolism. As part of the Guided Open Access pilot, editors from all 3 journals have discussed the reviewer reports and the manuscript's suitability for our journals. After careful evaluation, our editorial recommendation is to revise the manuscript and submit back through the Guided Open Access submission portal for consideration at *Nature Communications*. Provided the revisions satisfy all technical and editorial concerns, *Communications Biology* is also interested in publishing your manuscript. Please see details in the editorial assessment report in the pages below.

In brief, for publication in *Nature Communications*, major revisions are required to assess how MLCs cause mitochondrial fission, the kinetic properties of your method, why the OCR changes on illumination, to perform the analysis on more cells and to assess whether mitochondrial cristae are formed after light exposure in SLC25A46-/- cells. Alternatively, for publication in *Communications Biology*, major revisions would be required regarding how mitochondrial fission is caused by MLCs, an expansion of the number of cells analysed and confirmation of the localisation of LAMP2-mCherry-CRY2 on lysosomes, as well as further controls stipulated by the reviewers.

Please note that the Editorial Assessment Report is a standalone document that contains an editorial summary, recommendation and portable peer review reports. We have also included information about data accessibility and reproducibility, which we hope you find useful. As well as the EAR we have attached a marked up Reporting Summary.

If you have any questions about the recommended journals or would like advice on the revisions, you can contact me directly and I will be happy to assist. We look forward to receiving the revised version of your manuscript.

Yours sincerely,
Cara Eldridge
--
Dr Cara Eldridge
Associate Editor
Nature Communications

On behalf of the Guided OA editorial team

Contents of this Report

- **Manuscript overview**: details about your manuscript and the editorial team.
- **Manuscript assessment**: personalised recommendation from the editors.
- **About the editorial process**: an overview of the Guided Open Access process.
- **Annotated reviewer comments**: the referee reports with comments from the editors.
- **Open research evaluation**: advice for adhering to best reproducibility practices.

Manuscript overview

Manuscript number	Submission date	Decision date
GUIDEDOA-21-00015	22 January 2021	5 March 2021

**Title**	Light-activated mitochondrial fission through optogenetic control of mitochondria-lysosome contacts	**Corresponding author**	Jiajie Diao<br>ORCID: 0000-0003-4288-3203
**Preprint information**	No preprint found	**Peer review type**	Single-blind
**Editorial Assessment Team**	**Primary editor: Cara Eldridge**<br>**Home Journal:** *Nature Communications*, ORCID: 0000-0001-7001-2312<br>**Editorial team members:**<br>Nina Vogt, Nature Methods, ORCID: 0000-0002-2658-9138<br>Eve Rogers, Communications Biology, ORCID: 0000-0002-1841-7942		
**About your primary editor**	Cara joined Nature Communications in March 2020. After conducting her undergraduate and Master's degrees in Natural Sciences at the University of Cambridge, she went on to complete a PhD at the MRC-Laboratory of Molecular Biology and continued there as a postdoctoral researcher. The focus of her research has been on the effect of replication impediments on stem cell differentiation, and as part of this work she established human embryonic stem cells and induced pluripotent stem cells into the lab. She handles manuscripts in the areas of cellular biotechnology and methods, as well as imaging. Cara is based in the London office.		

Manuscript assessment and recommendation

**Editor's summary of the manuscript**	Here the authors report an optogenetics method to control mitochondrial dynamics through regulation of mitochondria-lysosome contacts (MLCs). These contacts are achieved by recruitment of the CRY2 and CIB proteins to lysosomes and mitochondria respectively, to obtain heterodimerisation under blue light illumination.<br><br>The reviewers found this method interesting but had a number of concerns which need to be addressed in a revised manuscript. The reviewers ask for mechanistic insight into how light-dependent MLCs cause mitochondrial fission, and understanding of the kinetics of this process. They also ask that you show that your method can be used for spatial as well as temporal precision. They have questions regarding the data that blue light alone appears to affect the OCR. The reviewers also for further data regarding the *SLC25A46*-/- cell experiments (please see full reports below).<br><br>There are also a number of control experiments and further statistical analyses required to improve your manuscript.

Editorial assessment overview

Nature Methods	**Not feasible to meet the editorial requirements**	*Nature Methods* considers the work described in this manuscript as an application of the CRY-CIB system, rather than a new method, which makes a revision of the manuscript unsuitable for the journal.
Nature Communications	**Major revisions needed**	*Nature Communications* would like to consider your manuscript further after major revisions. We are satisfied with the novelty of your work but request that you address all reviewer points.
Communications Biology	**Major revisions needed**	*Communications Biology* is also interested in publishing your manuscript. We would still require some, but not all, of the major revisions stipulated by the reviewers.

**Editor's recommendation**	***Option 1*: Revise for consideration at Nature Communications**<br>Our top suggestion would be to revise your manuscript and resubmit to *Nature Communications* for a second round of review.<br><br>***Option 2*: Revise for consideration at Communications Biology**<br>It would also be possible to revise your manuscript and resubmit to *Communications Biology* before being sent back for the reviewer's consideration.
**Next steps**	If you would like to follow our recommendation, when you are ready you can upload the revised manuscript, along with your point-by-point response to the reviewer's reports and editorial advice **here***. Please include the name of the journal you are resubmitting to in your cover letter.<br><br>*This URL links to your confidential home page and associated information about manuscripts you may have submitted, or that you are reviewing for us.

About the editorial process

By selecting the Nature Portfolio Guided Open Access option, your manuscript was assessed for suitability in three of our titles that provide venues for publication of high-quality work across the spectrum of methods development research: *Nature Methods, Nature Communications*, and *Communications Biology*. For more information about Guided Open Access, please see here.

Collaborative editorial assessment

Your editorial team discussed the manuscript to determine its suitability for the Nature Portfolio Guided OA pilot. Our assessment of your manuscript takes into account several factors, including whether the work meets the **technical standard** of the Nature Portfolio and whether the findings are of **immediate significance** to the readership of at least one of the participating journals in the Nature Portfolio Guided Open Access methods cluster.

Peer review

Experts were asked to evaluate the following aspects of your manuscript:

Novelty in comparison to prior publications;

Likely audience of researchers in terms of broad fields of study and size;

Potential impact of the study on the immediate or wider research field;

Evidence for the claims and whether additional experiments or analyses could feasibly strengthen the evidence;

Methodological detail and whether the manuscript is reproducible as written;

Appropriateness of the literature review.

Editorial evaluation of reviews

Your editorial team discussed the potential suitability of your manuscript for each of the participating journals. They then discussed the revisions necessary in order for the work to be published, keeping each journal's specific editorial criteria in mind.

Journals in the Nature portfolio will support authors wishing to transfer their reviews and (where reviewers agree) the reviewers' identities to journals outside of Springer Nature. For any questions about review portability, please contact our editorial office: guidedoa@nature.com

Annotated Reviewer Reports

**Reviewer #1**<br>*Report received: 17 February 2021*	
**Reviewer #1**	This reviewer has not chosen to waive anonymity. The reviewer's identity can only be shared with representatives of an established journal editorial office.
**Reviewer #1 expertise**<br>*Summarised by the editor*	Optogenetics for control of organelle location; Organelle membrane contact sites
**Editor's comments about this review**	This reviewer felt that the optogenetic tool described in your manuscript will be very useful but raises a number of concerns which must be addressed. They suggest major revisions will be required before publication.
**Reviewer #1 comments**	
**Overview**	The manuscript by Qiu et al. described the development of an optogenetic tool to control the mitochondrion-lysosome contacts (MLCs). The authors further applied this CRY2-CIB based tool to manipulate mitochondrial fission. As a proof of concept demo, the authors illustrated the use of their tools to partially rescue the mitochondrial function in SLC25A46-/- cells. It will be a useful tool for probing inter-organellar membrane contact sites and interrogate mitochondria function for the cell biology field. I support the publication of this work after a major revision, addressing concerns outlined below.<br><br>It will be a useful tool for probing inter-organellar membrane contact sites and interrogate mitochondria function for the cell biology field, but seems to be incremental at its current format without deeper mechanistic probing with the tool.

Specific comments

#	Reviewer comment	Editorial comment
**1**	The authors used the light-induced heterodimerization system made of CRY2 and CIB to control mitochondrion-	*Nature Communications ask for further insight into why MLCs*

	lysosome contacts. But why the light-dependent MLCs can cause the mitochondrial fission has not been discussed at all in the manuscript. More discussions or additional data should be added to fill this gap. How long does mitochondria fission take? Does the time scale of morphological changes align well with the kinetic properties of the CRY2-CIB optogenetic system?	*cause fission, and to see how fission corresponds with the timescale of your method.*<br><br>*Communications Biology ask that these points on mitochondrial fission be addressed in the revised manuscript.*
**2**	"SLC25A46 mutations can cause childhood-onset optic atrophy, axonal neuropathy……" Is there any correlation or causation between SLC25A46 mutations and mitochondrial fission? On Page 3, the authors mentioned that DRP1 and MFN are involved in mitochondria fission and fusion to cause human diseases. How about SLC25A46? It would be useful if the authors give a more detailed introduction regarding the connection between SLC25A46 and mitochondrial fission.	*Nature Communications ask for this discussion.*<br><br>*Communications Biology also ask for this discussion.*
**3**	It seems that images in the figures representing dark and light treatment conditions were not from the same cell. Given the high temporal resolution, it is most ideal to use the same cell for the mitochondrial fission demonstration (e.g., dark and different light stimulation time points). Representative movies showing the mitochondrial fission process should be provided. Admittedly, it may take at least 10 s to require one SIM image, but it is still possible to acquire images and quantitative data from the same cell. In the dark panel, one can still see some overlay between mitochondria and lysosome. Is this due to random collision? Or due to the dark activation of the system?	*Nature Communications suggest that you take images from the same cell as suggested if possible.*<br><br>*Communications Biology would also find it beneficial if it would be possible to take images from the same cell, but ask that this is discussed at minimum.*
**4**	The authors emphasized that they developed a spatiotemporal and reversible method to control mitochondrial fission. However, throughout the whole manuscript, there is no evidence to show that the new method can manipulate mitochondrial fission with spatial precision. new experiments should include the spatial control aspects using different cells in the same imaging field or in a subcellular region within the same cell.	*Nature Communications request that you show spatial control.*<br><br>*Communications Biology recommend including evidence of spatial precision of the new method if possible.*
**5**	The mitochondrial fission is caused by MLC as described by the authors in this manuscript. Several controls should be induced to strengthen this conclusion.	*Nature Communications ask that you perform these control experiments to strengthen the*

	a. Change the position of light sensors, put the CIB on lysosome and CRY2 with TOM20 to see If the same scenario can be visualized.<br><br>b. If the process is totally driven by heterodimerization, then other light-responsive heterodimerization systems (TULIP or iLID or eMag/pMag) with faster kinetics might do the same trick. The authors are encouraged to compare the efficacy of these optical dimerizers. Will the homo-oligomerisation of CRY2 alone affect the mitochondria fission? I strongly recommend the authors to co-transfect mCh-CRY2 and TOM20-CIBN-GFP as negative control.<br><br>c. Please use 3-color labeling in the same cell for LAMP-mCh-CRY2, TOM20-CIB-GFP and Mito (maybe blue color mito-tracker) to demonstrate light-dependent fission. Using mito-tracker instead of TOM20-CIB-GFP to show the morphology change of mitochondria. Since there is interaction between TOM20-CIB-GFP and LAMP-mCh-CRY2, so TOM20-CIB-GFP may not be suitable for monitoring mitochondrial dynamics here.	*results.*<br><br>*Communications Biology also request that you perform the stated control experiments.*
**6**	A major concern on the image organization and quantification. The resolution of SIM is around 100-200 nm. The size of mitochondria is in the range of 500-1000 nm. The gap in the membrane contact site probably spans 10-100 nm. How can the authors measure one single mitochondrion for quantification (page 7, Figure 2)? To mitigate this resolution issue, the authors are suggested to perform EM experiments to obtain more quantitative and accurate data.	*Nature Communications suggest performing EM experiments but understand this may not be possible.*<br><br>*Communications Biology ask that you discuss this concern regarding image organization and quantification at minimum.*
**7**	The authors should provide a critical evaluation on the strengths and weaknesses of the new tool described in the study compared to other similar ones in the literatures.	*Both Nature Communications and Communications Biology ask that you include this discussion.*
**8**	Multiple errors and typos noted, speaking for the poor quality of writing. The manuscript requires significant editing to correct a number of inaccuracies, omissions, and grammatical errors. There are too many examples to	*Both Nature Communications and Communications Biology ask that you address the required text changes.*

	give an exhaustive list, but some examples are given below.<br><br>a. Page 2, in Abstract, is it mitochondrion lysosome contacts (MLCs) or mitochondria-lysosome contacts? The authors used mitochondria in the title and the main text, but mitochondrion in the abstract. Please be consistent.<br>b. Page 2, MLCs or MLC, please make them consistent throughout the manuscript<br>c. Page 2, Page 5……, CIB or CIBN please make the term consistent throughout the manuscript and figures/figure legends.<br>d. Page 5, CaaX or CAAX? All the abbreviations should be double checked and displayed in a proper way.<br>e. Page 6, define the full name of abbreviation when it first appeared, e.g. TOM20 and LAMP<br>f. Page 1 "J-.L.G." should be J.L.G.<br>g. The sample size ("n" value) was missing for some key experiments (Figure 1, figure 2). Please add the "n" numbers.<br>h. Scale bar is missing (Figure S16) or duplicated (Figure 1b, Figure S3, S4, S5, S7, S10b, and S13b) in the related figures.<br>i. All the figure legends should be revised, especially for supplementary figures. Many key points are missing in these legends, including scale bar, experiment number, power or density of the light, statistical methods, and the related constructs used for transfection.<br>j. "Control" or "dark"? Figure 1b, 2c, 3a, Figure S4, S7, S9-12, S14<br>k. Multiple cell lines are used in this study. A table is recommended to summarize the quantification data for each cell type.	

**Reviewer #2**<br>*Report received: 20 February 2021*	
**Reviewer #2**	This reviewer has not chosen to waive anonymity. The reviewer's identity can only be shared with representatives of an established journal editorial office.
**Reviewer #2 expertise**<br>*Summarised by the editor*	Organelle maintenance; Microscopy methods including structured illumination microscopy
**Editor's comments about this review**	This reviewer found your work interesting but asks for additional experimental data and controls to support that this optogenetic system induces mitochondrial fission.
**Reviewer #2 comments**	
**Overview**	In this article, Qiu et al. describe an optogenetic method to reversibly induce mitochondria-lysosome contacts and modulate mitochondrial dynamics. They use a previously described dimerization technique involving the heterodimerization of CRY2 with CIB under blue light. The authors use a lysosomal anchored CRY2 and a mitochondrial outer membrane-anchored CIB, allowing them to force mitochondria-lysosome contacts under blue light. Photoactivation of contact between the two organelles results in a reversible change in the mitochondrial morphology network. Increasing lysosome-mitochondria contacts by this system induces a quantifiable fragmentation of the mitochondrial network, suggesting that it promotes mitochondrial fission. Finally, the authors use this system in SLC25A46 -/- cells characterized by a hyperfused mitochondrial network and a decreased oxygen consumption rate (OCR) to rescue these parameters. The forced mitochondria-lysosome contacts caused mitochondrial fragmentation and partially increased the OCR in these cells suggesting this system could partially rescue the mitochondrial dysfunctions in the absence of SLC25A46.<br><br>Overall, the data presented in this manuscript convincingly demonstrate that the CRY2-CIB dimerization system allows for reversible modulation of mitochondria-lysosome contacts, which can be used to modulate mitochondrial morphology. However, the concept of using optogenetics for contact induction is not particularly novel as a similar system was recently described that allowed an acute manipulation of organelle contacts, notably between mitochondria and lysosomes (Benedetti et al., 2020. doi: 10.7554/eLife.63230). Additionally, some experimental data and controls

	are further required to clearly support that this optogenetic system induces mitochondrial fission and that it could be used to rescue mitochondrial dysfunction in SLC25A46 -/- cells.

Specific comments

#	Reviewer comment	Editorial comment
**1**	MAJOR: It is not clear whether forcing lysosome-mitochondria contact using their optogenetic approach directly promotes mitochondrial fission, as it is equally possible that it may be decreasing mitochondria fission. To determine fission, the authors compared the morphology of mitochondria before and after the induction of contact. Using morphological changes as the sole parameter is problematic as an increase in fragmented mitochondria or round mitochondria can occur either by increasing fission or a decrease in fusion. Measuring the mitochondrial fission rate before and after dimerization would strengthen their conclusion that their photoactivation-induced contact is causing mitochondrial fission vs. fusion. Additionally, it has been shown that lysosome-mitochondria contacts are involved in the mitochondrial division and the regulation of the function of both mitochondria and lysosomes. Thus, it is very likely that forcing contacts between these two organelles is affecting both mitochondrial homeostasis and lysosome biology, which may indirectly cause mitochondria fragmentation. The authors should address these possibilities.	*Nature Communications ask that you look at how MLCs induce fission (as requested by Reviewer #1).*<br><br>*Communications Biology see the benefit in measuring mitochondrial fission rate before and after dimerization and request that further information on how MLCs induce fission be provided if possible.*
**2**	MAJOR: The experiments testing mitochondria function restoration in the SLC25A46-/- cells using CRY2-CIB are not convincing, given that simply expressing CRY2 and CIB constructs have a negative effect on the OCR by itself. Similarly, blue light emission alone seems to affect the OCR. For this reason, although inducing morphology change in SLC25A46 -/- cells do appear to increase the OCR, it is not clear whether the changes induced are a true “rescue.” Are the changes in OCR induced by blue light emission and/or forced lysosome-mitochondria contacts are reversible? The changes may induce detrimental alterations of mitochondrial functions. An excessive fragmentation and increased OCR have been	*Nature Communications ask that you address the reason for the OCR changes on blue light emission and to address whether these changes are reversible.*<br><br>*Communications Biology requests that this concern be addressed too, or discussed at minimum.*

	linked to mitochondrial dysfunctions. Showing that the OCR changes induced are reversible would strengthen the conclusion that the system can potentially rescue mitochondrial dysfunctions in the cell used.	
**3**	MINOR: The authors should state in the text that the LAMP construct encodes for Lamp1 to avoid confusion with Lamp2.	*Both Nature Communications and Communications Biology ask that you make the requested change.*
**4**	MINOR: Sup Fig 3: it is unclear which parameters were measured to determine the best concentration of use. Is the ng of the plasmid total plasmid or the CYR2 plasmid? This should be indicated in the text or legend.	*Both Nature Communications and Communications Biology ask that you make the requested change.*
**5**	MINOR: Fig1e: It would be nice to measure the contacts between lysosome and mitochondria after blue light emission was stopped to show whether this is truly reversible	*Nature Communications would like the reversibility addressed.*<br><br>*Communications Biology would also like this addressed.*
**6**	MINOR: Line 122 to 124: This should be removed since it is expected that the lysosome does not leave the mitochondria-lysosome contact after division since the cells are under blue light emission, which forces lysosomes-mitochondria to contact.	*Both Nature Communications and Communications Biology ask that you make the requested change.*
**7**	MINOR: Genes name should be italicized	*Both Nature Communications and Communications Biology ask that you make the requested change.*
**8**	MINOR: Is there some evidence that mitochondria contacts are decreased/altered in SLC25A46-/- cells?	*Nature Communications ask that you address this.*<br><br>*Communications Biology ask that you either provide evidence or discuss this point in the revised manuscript.*
**9**	MINOR: Fig3 e and g: The labeling of the two conditions is not clear. Why is the first role of the X-axis labeled as “SLC -/- vs. SLC -/- light.” It is unclear what is being analyzed here. Is this the difference between the two conditions?	*Both Nature Communications and Communications Biology ask that you make the requested change.*
**10**	MINOR: Line 193 to 195. I find this sentence quite confusing. It is not clear what the authors mean by “not full view,” for instance.	*Both Nature Communications and Communications Biology ask that you make the requested change.*

**11**	MINOR: Line 224: The statement “affords a viable method for treating this type of mitochondrial disorder” is too strong of a statement given the data presented. It should be removed or written less affirmatively.	*Both Nature Communications and Communications Biology ask that you make the requested change.*
**12**	The number of cells imaged per experiment seems extremely small. In most experiments, it is written that 6 cells were imaged (n = 6). This is too small to perform such analysis, especially considering that mitochondrial morphology is quite variable within the same population of cells (e.g., Hela Cells). In the report summary, it is written that the number of cells comes from at least 3 independent experiments. Does it mean that only two cells were imaged by trial for a total of 6 cells (in which case the number of cells needs to be increased) or that only one trial is shown in the figures (in which cases all cells imaged from all trials should be used for analysis)? The authors need to clarify this, and they should also increase their n.	*Nature Communications ask that you address the statistical and reproducibility concerns raised by this reviewer.*<br><br>*Communications Biology also ask that you address the concerns regarding statistics and reproducibility.*
**13**	The authors should also consider using “SuperPlots” to demonstrate the robustness of their experiments and the cell-to-cell variability both of which are bound to exist in a transient transfected experimental system.	*Nature Communications agree that this would be useful.*<br><br>*Communications Biology would find this beneficial but not strictly necessary.*

**Reviewer #3**<br>*Report received: 2 March 2021*	
**Reviewer #3**	This reviewer has not chosen to waive anonymity. The reviewer's identity can only be shared with representatives of an established journal editorial office.
**Reviewer #3 expertise**<br>*Summarised by the editor*	Mitochondrial dynamics; Metabolism; Lysosome contacts; SLC25A46
**Editor's comments about this review**	This reviewer found your method for optogenetic control of mitochondrial dynamics novel but requests further work showing why mitochondria-lysosome contacts induce fission.
**Reviewer #3 comments**	
**Overview**	Mitochondrial fusion/fission dynamics plays important roles in the metabolic functions and cellular homeostasis. The authors in this study developed a method to control mitochondrial fission using optogenetic mitochondria-lysosome contacts (MLCs) systems. Furthermore, the authors showed that this system facilitates mitochondria fission in SLC25A46-/- cells, which then restores several mitochondrial functions. Although the optogenetic control of mitochondrial dynamics is novel, the authors need to show why MLC induces mitochondrial fission and need to explain how they quantify different mitochondrial morphologies in more detail before the acceptance of the manuscript. In addition, previous results showed that loss of SLC25A46 causes disruption of mitochondrial cristae and destabilization of the MICOS complex. Therefore, the authors should see mitochondrial cristae and MICOS complex after a light exposure in SLC25A46-/- cells.

Specific comments

#	Reviewer comment	Editorial comment
1	Why does the physical contact between mitochondria and lysosomes induce mitochondrial fission? Is mitochondrial fission by the authors' optogenetic MLC system a DRP1-dependent or a RAB7-dependent?	*Nature Communications ask you to address this – as Reviewer #1 and #2.*<br><br>*Communications Biology also request that the requested clarifications are addressed.*

**2**	Fig1b: The author should confirm that LAMP2-mCherry-CRY2 localizes on lysosomes.<br>The number of lysosomes detected by LAMP-mCherry-CRY2 seems to be too small, and after light illumination, the number increased. Why?	*Nature Communications ask that you address the LAMP2-mCherry-CRY2 localisation.*<br><br>*Communications Biology also ask that you address the localization of LAMP2-mCherry-CRY2 on lysosomes.*
**3**	How did authors quantify mitochondrial morphology and calculate the size of mitochondria? The authors should state the detailed method in the materials and methods section.	*Nature Communications ask that you are clear regarding this point.*<br><br>*Communications Biology also ask that the detailed method be provided.*
**4**	Fig3: The reduction of mitochondrial hyperfusion by optogenetic MLC in SLC25A46-/- cells is interesting. Is this specific in SLC25A46-/- cells? Does mitochondrial fission by the optogenetic MLC also occur in DRP1-deficient or MFF/MiD49/MiD51-deficient cells? In addition, the authors should see mitochondrial cristae and MICOS complex after a light exposure in SLC25A46-/- cells.	*Nature Communications ask that you address this point.*<br><br>*Communications Biology request that this point is addressed or further discussed.*
**5**	Fig3c: Why are the initial OCR scores in SLC-/- and SLC-/- +light so different?	*Nature Communications ask that you address this question brought up by Reviewer #2 and #3.*<br><br>*Communications Biology also request that this point be addressed.*
**6**	Fig3e and g: What does SLC-/- vs SLC-/- light mean?	*Both Nature Communications and Communications Biology ask that you make the requested change.*
**7**	MINOR:<br>a) TOM20 should be TOMM20.<br>b) Fig2a legend: grey arrows should be white arrows.	*Both Nature Communications and Communications Biology ask that you make the requested change.*

Open Research Evaluation	
**Data Availability**	
**Data Availability Statement**	The Nature Portfolio journals strongly support public availability of data and custom code associated with the paper in a persistent repository where they can be freely and enduringly accessed or as a supplementary data file when no appropriate repository is available. If data and code can only be shared on request, please explain why in your data Availability Statement, and also in the correspondence with your editor. For more information, please refer to https://www.nature.com/nature-research/editorial-policies/reporting-standards#availability-of-data<br><br>See our policy page for more details.
**Recommended data deposition**	Nature Portfolio policies include a strong preference for research data to be archived in public repositories. For data types without specific repositories, we recommend that data are deposited in a generalist repository such as figshare or Dryad. If you need help complying with this policy, or need help depositing and curating your research data (including raw and processed data, text, video, audio and images) you should consider:<br>• Contacting Springer Nature's Research Data Helpdesk for advice,<br>• finding a suitable data repository for your data,<br>• Or uploading your data to Springer Nature's Research Data Support service.<br><br>Please note there are fees for using Springer Nature's Research Data Support service.
**Materials availability**	We suggest that the constructs generated in your manuscript are made available via Addgene and/or the sequences are made public.
**Source data**	The following figure panels should be accompanied by the underlying source data: **Main figures 1e, 2e,g, 3b-g** and **Supplementary figures 2a-c, 7c, 9b, 10c-d, 11b, 12b, 13a, 18.**

	Source data files will be mandatory prior to publication in a Nature Portfolio journal.
**Data citation**	Where datasets are hosted in public repositories that provide datasets with Digital Object Identifiers (DOIs), we encourage these datasets to be formally cited in reference lists. Citations of datasets, when they appear in the reference list, should include the minimum information recommended by DataCite and follow journal style. For example: Hao, Z., AghaKouchak, A., Nakhjiri, N., Farahmand, A. Global Integrated Drought Monitoring and Prediction System (GIDMaPS) Data sets. *figshare*. http://dx.doi.org/10.6084/m9.figshare.853801 (2014).<br><br>Citing and referencing data in publications supports reproducible research, by increasing the transparency and provenance tracking of data generated or analysed during research. Citing data formally in reference lists also helps facilitate the tracking of data reuse and may help assign credit for individuals' contributions to research. A number of Springer Nature imprints are signatories of the Joint Declaration on Data Citation Principles, which stress the importance of data resources in scientific communication.
**Research ethics**	
**Competing Interests**	In the interests of transparency and to help readers form their own judgements of potential bias, Nature Portfolio journals require authors to declare any competing financial and/or non-financial interests in relation to the work described.<br><br>Please provide a 'Competing interests' statement using one of the following standard sentences:<br>• The authors declare the following competing interests: [specify competing interests]<br>• The authors declare no competing interests.<br><br>See our competing interests policy for further information: https://www.nature.com/nature-research/editorial-policies/competing-interests

<table>
<tr><th colspan="2">Methods assessment and reproducibility</th></tr>
<tr><td>Statistical Evaluation</td><td>
Wherever statistics have been derived (e.g. error bars, box plots, statistical significance) the legend needs to provide and define the n number (i.e. the sample size used to derive statistics) as a precise value (not a range), using the wording “n=X biologically independent samples/animals/cells/independent experiments/n= X cells examined over Y independent experiments” etc. as applicable.
<u>Legends requiring revision:</u>

Please note that this information is missing in the legends of figures 3b-g and supplementary figure 13a.
Although ‘n’ is provided, please describe the nature of entity for ‘n’ in the figures 1e; 2e, g and supplementary figures 2a-c; 7c; 9b; 10c, d; 11b; 12b; 18.

Statistics such as error bars, significance and p values cannot be derived from n<3 and must be removed from all such cases.
We strongly discourage deriving statistics from technical replicates, unless there is a clear scientific justification for why providing this information is important. Conflating technical and biological variability, e.g., by pooling technically replicates samples across independent experiments is strongly discouraged. (For examples of expected description of statistics in figure legends, please see the following https://www.nature.com/articles/s41467-019-11636-5 or https://www.nature.com/articles/s41467-019-11510-4).
All error bars need to be defined in the legends (e.g. SD, SEM) together with a measure of centre (e.g. mean, median). For example, the legends should state something along the lines of “Data are presented as mean values +/- SEM” as appropriate.
<u>Legends requiring revision:</u>

Please note that the error bars need to be defined in the legend of supplementary figure 13a.

</td></tr>
</table>

**Figure legends**	The figure legends must indicate the statistical test used. Where appropriate, please indicate in the figure legends whether the statistical tests were one-sided or two-sided and whether adjustments were made for multiple comparisons.<br>For null hypothesis testing, please indicate the test statistic (e.g. F, t, r) with confidence intervals, effect sizes, degrees of freedom and P values noted.<br>Please provide the test results (e.g. P values) as exact values whenever possible and with confidence intervals noted.<br>**Legends requiring revision:**<br>1. Please indicate the statistical test used for data analysis and where appropriate, please specify whether it was one-sided or two-sided and whether adjustments were made for multiple comparisons, in the legends of figures 1e; 2e, g; 3b, d-g and supplementary figures 7c; 12b.<br>2. Please note that the exact p value should be provided, when possible, in the legends of figures 1e; 2e, g; 3b, d-g and supplementary figures 7c; 12b.
**Reproducibility**	Please state in the legends how many times each experiment was repeated independently with similar results. This is needed for all experiments, but is particularly important wherever results from representative experiments (such as micrographs) are shown. If space in the legends is limiting, this information can be included in a section titled “Statistics and Reproducibility” in the methods section.<br>**Legends requiring revision:**<br>Please note that this information is missing in the legends of figures 1b; 2c, h-j and supplementary figures 1; 3; 5a-c; 6a-d; 13b.
**Methods Descriptions**	The Methods must contain sufficient detail such that the work could be repeated. It is preferable that all key methods be included in the main manuscript, rather than in the Supplementary Information.<br>Nature Portfolio journals encourage authors to share their step-by-step experimental protocols on a protocol sharing

	platform of their choice. Where such protocols are available, please provide a DOI or other citation details in the paper. The Nature Portfolio's *Protocol Exchange* is a free-to-use and open resource for protocols; protocols deposited in *Protocol Exchange* are citable and can be linked from the published article. More details can found at www.nature.com/protocolexchange/about
**Data presentation**	Please ensure that data presented in a plot, chart or other visual representation format shows data distribution clearly (e.g. dot plots, box-and-whisker plots). When using bar charts, please overlay the corresponding data points (as dot plots) whenever possible and always for n ≤ 10. (Please see the following editorial for the rationale behind this request and an example https://www.nature.com/articles/s41551-017-0079).
**Study design and reporting**	
**Sample size calculation**	For live cell imaging studies, please elaborate on how sample sizes were chosen (providing relevant citations if available).<br>Please describe how sample size was determined for experiments other than live cell imaging studies as well, detailing any statistical methods used to predetermine sample size OR if no sample size calculation was performed, describe how sample sizes were chosen and provide a rationale for why these sample sizes are sufficient.
**Blinding**	Please specify the experiments where blinding was not possible. Also, please provide a rationale as to why investigators were not blinded during those experiments where blinding was not possible.
